# Mechanistic insights into probiotic modulation of the gut–immune axis and their role as sustainable antibiotic alternatives in poultry production: An integrative review

**DOI:** 10.14202/vetworld.2026.3039-3080

**Published:** 2026-07-17

**Authors:** Andreas Berny Yulianto, Aswin Rafif Khairullah, Widya Paramita Lokapirnasari, Mohammad Anam Al-Arif, Zulfi Nur Amrina Rosyada, Emy Koestanti Sabdoningrum, Bodhi Agustono, Mirni Lamid, Kartika Purnamasari, Bima Putra Pratama, Riza Zainuddin Ahmad, Wasito Wasito, Saifur Rehman, Muhammad Aviv Firdaus

**Affiliations:** 1 Faculty of Veterinary Medicine, Universitas Wijaya Kusuma Surabaya, Jl. Dukuh Kupang XXV No. 54, Dukuh Kupang, Dukuh Pakis, Surabaya 60225, East Java Indonesia.; 2 Research Center for Veterinary Science, National Research and Innovation Agency (BRIN), Jl. Raya Bogor Km. 46 Cibinong, Bogor 16911, West Java, Indonesia.; 3 Division of Animal Husbandry, Faculty of Veterinary Medicine, Universitas Airlangga, Jl. Mulyorejo, Kampus C Mulyorejo, Surabaya 60115, East Java, Indonesia.; 4 Faculty of Health, Medicine, and Life Sciences, Universitas Airlangga, Jl. Wijaya Kusuma No.113 Giri, Banyuwangi 68422, East Java, Indonesia.; 5 Research Center for Process Technology, National Research and Innovation Agency (BRIN), Serpong, South Tangerang 15314, Banten, Indonesia.; 6 Department of Pathobiology, Faculty of Veterinary and Animal Sciences, Gomal University, RV9W+GVJ, Indus HWY, Dera Ismail Khan 27000, Pakistan.; 7 Master Program of Veterinary Agribusiness, Faculty of Veterinary Medicine, Universitas Airlangga, Jl. Mulyorejo, Kampus C Mulyorejo, Surabaya 60115, East Java, Indonesia.

**Keywords:** antibiotic alternatives, disease resistance, gut health, immune modulation, microbiota, poultry, probiotics, sustainable production

## INTRODUCTION

The poultry immune system plays a central role in determining resistance to bacterial, viral, and parasitic infections while maintaining physiological stability and production efficiency [1]. It comprises coordinated innate defenses, such as macrophages, heterophils, and dendritic cells, and adaptive components involving B and T lymphocytes [2, 3]. These systems interact closely with gastrointestinal health and microbial composition, forming the gut–immune axis, which is now recognized as a key regulatory network in modern poultry production [4]. Increasing evidence indicates that immune competence is not merely a defensive mechanism but a major determinant of resilience, productivity, and sustainability in intensive farming systems [5].

The poultry industry faces persistent challenges that compromise immune stability, including pathogen pressure, environmental stress, and management constraints [6, 7]. Historically, antibiotic growth promoters (AGPs) have been used to sustain productivity. However, their extensive application has raised global concerns regarding antimicrobial resistance (AMR), drug residues in animal products, and public health risks [8]. As regulatory restrictions on AGPs intensify, the development of safe and sustainable alternatives has become imperative [9]. In this context, strengthening immune regulation, rather than simply accelerating growth rate, has emerged as a strategic priority for long-term productivity and disease control [10].

Probiotics have gained considerable attention as functional feed additives that modulate gut microbial ecology and host physiology [11, 12]. Strains such as *Lactobacillus* spp., *Bifidobacterium* spp., and *Bacillus* spp. have been associated with enhanced innate immune activity, lymphocyte proliferation, improved mucosal (IgA) and systemic (IgY) antibody responses, and reinforced intestinal barrier integrity [13, 14]. Through competitive exclusion, antimicrobial metabolite production, and stimulation of mucosal immunity, probiotics contribute to inhibiting pathogens and maintaining intestinal homeostasis [15]. These multifaceted interactions highlight their potential to reinforce the gut–immune axis.

Although numerous reviews have discussed probiotic supplementation in poultry, most have primarily emphasized growth performance, feed conversion efficiency, or general health indicators. Such approaches often treat immune responses as secondary observations rather than central mechanistic drivers. In contrast, this review positions immune modulation as the principal analytical framework. Growth enhancement is interpreted as a downstream outcome of optimized immune–microbiota interactions, rather than the primary endpoint [8]. By integrating molecular, cellular, and organ-level evidence, this review synthesizes mechanistic insights into cytokine regulation, immune cell activation, gut-associated lymphoid tissue (GALT) dynamics, lymphoid organ development, oxidative stress modulation, and post-vaccination immune responsiveness.

Despite promising findings, significant inconsistencies persist in the literature due to strain-specific variability, differences in dosage and supplementation duration, heterogeneity in experimental designs, and limited long-term commercial validation [16–18]. Most existing reviews address probiotics from a performance-oriented perspective, with limited integration of detailed immunological mechanisms, including effects on GALT, lymphoid organ development, cytokine networks, oxidative stress pathways, and vaccine responsiveness. There is a clear need for a focused, mechanism-driven synthesis that bridges molecular and cellular insights with practical translational outcomes in commercial poultry systems. Such a synthesis is essential for resolving conflicting results and providing evidence-based guidance for probiotic applications as sustainable AGP alternatives [19, 20].

Accordingly, this review aims to elucidate the mechanistic pathways through which probiotics regulate innate and adaptive immune responses in poultry; analyze their effects on GALT and lymphoid organ modulation; evaluate their influence on post-vaccination immunity and pathogen resistance; examine molecular signaling interactions within the gut–immune axis, including cytokine networks and oxidative stress regulation; and assess their role as sustainable alternatives to AGPs from an immunological and translational perspective. By synthesizing current evidence and highlighting knowledge gaps, this review aims to provide a comprehensive framework to support the development of targeted, immune-centric probiotic strategies to improve poultry health, productivity, and sustainability.

## REVIEW METHODOLOGY

The literature search encompassed publications from January 2000 to March 2025. No specific geographic restriction was applied; however, studies were drawn from global research to capture broad applicability across various poultry production systems.

This study was conducted as an integrative review to synthesize current evidence on the mechanistic roles of probiotics in modulating the gut–immune axis and their application as sustainable alternatives to AGPs in poultry production. An integrative approach was chosen to incorporate diverse study designs, including experimental, mechanistic, and applied research, thereby providing a comprehensive perspective on immune modulation and practical implications.

### Literature search strategy

A structured literature search was performed using major scientific databases, including Scopus, Web of Science, and PubMed. The search employed combinations of keywords and Boolean operators, including “probiotics,” “poultry,” “gut–immune axis,” “immune modulation,” “intestinal morphology,” “vaccination response,” “oxidative stress,” and “antibiotic growth promoters” or “AGP replacement.” Reference lists of relevant reviews and primary studies were also screened to identify additional eligible articles.

### Study selection and data synthesis

Studies were included if they (i) investigated probiotic supplementation in poultry species (e.g., broilers, layers, breeders, or indigenous chickens), (ii) reported immunological, microbiological, molecular, or production-related outcomes, and (iii) were original research articles published in English-language peer-reviewed journals. Studies focusing exclusively on non-poultry species, lacking measurable immune or gut health parameters, or published as conference abstracts and non-peer-reviewed reports were excluded.

Relevant data extracted from each study included probiotic strain(s), dosage, supplementation duration, experimental design, immune parameters (e.g., cytokine expression, antibody titers, lymphoid organ indices), gut morphology, microbiota composition, and production performance indicators. The findings were synthesized thematically to evaluate mechanistic pathways, consistency of outcomes, strain-specific effects, dose–response relationships, and translational relevance to commercial poultry systems, while also identifying key research gaps and industry implications.

## BASIC CONCEPTS OF PROBIOTICS IN POULTRY

The use of probiotics in poultry nutrition and health strategies has gained popularity over the past two decades, driven by the growing need for non-antibiotic approaches to improve production performance and strengthen the immune system [21]. To comprehensively assess the role of probiotics, a basic understanding of their definition, the types of microorganisms used, and the biological mechanisms underlying their effects in the poultry digestive tract is essential [22].

## DEFINITION OF PROBIOTICS

Scientifically, probiotics are defined as live microorganisms that can provide health benefits to the host when consumed in adequate amounts [23]. Probiotics are defined as live microorganisms that, when administered in adequate amounts, confer health benefits on the host, and in poultry, they are primarily used to improve gut microbiota balance and modulate the avian immune system [24]. Thus, probiotics not only act as commensal microbes but are active organisms that have been experimentally shown to produce physiological effects on the host, such as modulating the immune system, improving intestinal function and integrity, and inhibiting the growth of pathogens [25].

Conceptually, the definition of probiotics encompasses several fundamental scientific aspects. First, probiotics must consist of living microorganisms, namely cells that can survive until they reach the digestive tract and continue their biological activity [26]. This viability is assessed by the microbe's ability to withstand extreme conditions, including gastric pH, bile salts, and other gastrointestinal conditions [27]. Second, the effects of probiotics are dose-dependent, so they must be administered in sufficient quantities [28]. Various studies have shown that the effective dose range is between 10⁶ and 10¹⁰ colony-forming units (CFU), depending on the strain and the intended use [29].

The third important aspect is that the benefits of probiotics are strain-specific [30]. This means that each strain of microorganism has different genetic characteristics, colonization abilities, and physiological effects [31]. Therefore, claims of probiotic effectiveness cannot be generalized at the genus or species level but must be proven for each strain [32]. Fourth, probiotics must have a verified safety profile, including not carrying potentially transferable AMR genes (ARGs), not producing toxins, and not exhibiting pathogenic properties [33]. This safety assessment refers to the Generally Recognized as Safe (GRAS) or Qualified Presumption of Safety (QPS) systems used in evaluating microorganisms for feed and food applications [34].

In the context of poultry, probiotics are defined as microorganisms that not only improve gastrointestinal health but also modulate the immune system, enhance vaccine responses, and suppress the colonization of pathogens such as *Salmonella*, *Escherichia coli*, and *Clostridium* spp. [35]. Thus, probiotics function as biological agents that help maintain gastrointestinal balance and enhance the performance of the poultry immune system through mechanisms involving interactions among the gut microbiota, the intestinal epithelium, and the GALT [36].

## TYPES AND SOURCES OF PROBIOTICS FOR POULTRY

A variety of microorganisms have been identified and used as probiotics in poultry for their biological properties, ability to colonize the intestinal tract, and physiological effects on the host [37]. Generally, probiotics for poultry are derived from groups such as lactic acid bacteria, spore-forming bacteria, commensal anaerobes, and several types of yeast, which are known for their high stability and safety [38]. Strains are selected through a rigorous process that includes testing for resistance to gastric acid and bile salts, adhesion to the intestinal epithelium, competition with pathogens, and potential modulation of the immune system [39]. However, beyond these biological criteria, commercial applicability in poultry production also depends on processing stability, shelf life, consistency of *in vivo* performance, and regulatory acceptance, which create important functional distinctions among probiotic groups [35].

The *Lactobacillus* spp. group is the most commonly used probiotic species in the poultry industry [40]. Species such as *Lactobacillus acidophilus*, *Lactobacillus plantarum*, and *Lactobacillus reuteri* are known to produce lactic acid, bacteriocins, and various bioactive metabolites that inhibit the growth of pathogenic bacteria and help maintain the balance of the gut microbiota [41]. Furthermore, these bacteria can enhance mucosal immune system activity by stimulating IgA production and regulating cytokine responses [42]. Notably, several *Lactobacillus* strains, particularly *L. acidophilus* and *L. plantarum*, are supported by substantial *in vivo* poultry studies demonstrating improvements in intestinal morphology and immune parameters. Nevertheless, their relatively lower resistance to high temperature feed pelletization and environmental stress may limit viability during industrial feed processing unless protective technologies (e.g., microencapsulation or post-pellet spraying) are applied [41].

Additionally, *Bacillus* spp., such as *Bacillus subtilis* and *Bacillus licheniformis*, are widely used as probiotics due to their ability to form spores that are highly resistant to high temperatures, environmental stress, and feed pelletization processes [43]. *Bacillus* strains can produce digestive enzymes, improve nutrient utilization, and provide immunomodulatory effects through macrophage activation and regulation of inflammatory responses [44]. The robustness of their spores makes this group highly superior as commercial probiotics, particularly in intensive poultry production systems [45]. These species are among the most extensively validated probiotics in commercial broiler and layer trials, with strong *in vivo* evidence supporting their effects on growth performance and feed efficiency. Compared with non-spore-forming bacteria, *Bacillus* spp. exhibit superior thermal resistance, storage stability, and survivability during feed manufacturing, making them particularly compatible with large-scale commercial poultry systems [44].

The *Bifidobacterium* spp. group, although more commonly found in mammals, has also been shown to be beneficial for birds, particularly in maintaining intestinal microbial balance and inhibiting the colonization of pathogenic bacteria [46]. *Bifidobacterium bifidum* and *Bifidobacterium animalis* contribute to the production of acetic acid and various other metabolites that help strengthen the integrity of the intestinal mucosal barrier [47]. However, compared to *Lactobacillus* and *Bacillus*, the evidence base in poultry remains more limited, and some functional claims are partially extrapolated from mammalian models. However, compared with *Lactobacillus* and *Bacillus*, poultry-specific *in vivo* data remain more limited, and some functional claims are partially extrapolated from mammalian models, which may reduce translational certainty under commercial poultry conditions [41, 43].

On the other hand, several *Enterococcus* species, including *Enterococcus faecium*, are used as probiotics due to their ability to rapidly colonize the digestive tract of poultry and efficiently compete with pathogens such as *Salmonella* and *E. coli* [48]. However, the use of these bacteria must be accompanied by a thorough safety assessment to ensure that they do not contain potentially transmitted ARGs [49]. This regulatory concern may restrict broader industry adoption despite demonstrated pathogen exclusion capacity.

In addition to bacterial groups, probiotics derived from yeast, such as *Saccharomyces boulardii*, also have significant potential to support poultry health [50]. This yeast is known to be resistant to various antibiotics and gastrointestinal conditions, and to suppress pathogen colonization through toxin-binding mechanisms, increase digestive enzyme activity, and modulate both inflammatory and anti-inflammatory immune responses [51]. While promising poultry studies exist, certain mechanistic insights, particularly for *S.* *boulardii, *are derived from non-avian models and require further confirmation in controlled *in vivo* poultry experiments. While promising, certain mechanistic interpretations of *S.* *boulardii* are based on non-avian studies and require further controlled validation in poultry [50].

From a critical comparative perspective, clear functional trade-offs emerge among probiotic groups. *Lactobacillus* spp. are strongly associated with enhanced mucosal and humoral immunity (particularly IgA stimulation and cytokine modulation), making them suitable for immune-oriented interventions [40]. *Bacillus* spp. combine moderate but consistent immunomodulatory effects with marked improvements in nutrient digestibility and feed conversion efficiency [43]. *Bifidobacterium* spp. primarily reinforce barrier integrity and microbiota stability, indirectly supporting immune resilience. *Enterococcus* spp. and *S.* *boulardii* emphasize pathogen exclusion but present either regulatory or evidentiary limitations [48].

Importantly, the dominance of *Bacillus* spp. in commercial poultry systems is largely driven by technological robustness and economic practicality rather than purely superior immunological potency. Spore formation allows survival during feed pelletization temperatures exceeding 80°C, extended storage without refrigeration, and stability under variable farm conditions [43]. This ensures predictable dosing and minimizes viability losses during distribution. Moreover, the enzyme-producing capacity of *Bacillus* strains directly enhances feed efficiency, an economically critical parameter in intensive broiler and layer production [44]. Combined with strong field validation and regulatory acceptance, these advantages explain why *Bacillus*-based probiotics currently dominate commercial poultry markets. To enhance comparative clarity, Table 1 summarizes the major probiotic groups by strain type, dominant immune effects, processing stability, and level of industrial application [40–51].

**Table 1:** Comparative characteristics of major probiotic groups used in poultry production.

**Probiotic group**	**Representative strains**	**Dominant immune and functional effects**	**Processing and gastrointestinal stability**	**Industry application level**	**References**
*Lactobacillus *spp.	*Lactobacillus acidophilus*, *Lactobacillus plantarum*, *Lactobacillus reuteri*	Strong mucosal immune stimulation through enhanced IgA production, cytokine modulation, improved intestinal morphology, and pathogen inhibition via lactic acid and bacteriocin production	Moderate heat resistance; relatively sensitive to feed pelletization and environmental stress unless protected by microencapsulation or post-pellet application	Widely used; strong *in vivo* poultry evidence and extensive experimental support	[40–42]
*Bacillus* spp.	*Bacillus subtilis*, *Bacillus licheniformis*	Moderate but consistent immune modulation, macrophage activation, cytokine balance, digestive enzyme production, improved nutrient utilization and feed efficiency	Very high stability due to spore formation; highly resistant to pelletization, storage, and farm environmental stress	Dominant in commercial poultry systems; extensive field validation and strong economic relevance	[43–45]
*Bifidobacterium* spp.	*Bifidobacterium bifidum*, *Bifidobacterium animalis*	Enhancement of intestinal barrier integrity, microbiota stabilization, acetic acid production, and indirect immune resilience support	Moderate gastrointestinal survival; lower thermal and processing stability compared with spore-forming bacteria	Limited to moderate use; fewer poultry-specific *in vivo* studies	[46, 47]
*Enterococcus *spp.	*Enterococcus faecium*	Rapid intestinal colonization, strong pathogen competition, exclusion of *Salmonella* spp. and *Escherichia coli*	Good gastrointestinal survival; requires strict safety and antimicrobial resistance screening	Moderate use; restricted by regulatory and biosafety concerns	[48, 49]
Yeast (*Saccharomyces boulardii*)	*S* *.* *boulardii*	Pathogen toxin binding, modulation of inflammatory and anti-inflammatory immune responses, improved digestive enzyme activity	High gastrointestinal resilience and good feed stability	Emerging use; promising but still variable poultry-specific evidence	[50, 51]

## STRAIN-SPECIFIC EFFECTS AND QUANTITATIVE EVIDENCE

Probiotic efficacy in poultry is widely acknowledged to be strain-specific; however, many published studies and narrative summaries tend to generalize effects at the genus level (e.g., *Lactobacillus* and *Bacillus*) without direct comparative synthesis.

Recent systematic reviews and meta-analyses (2024–2025) highlight substantial heterogeneity in outcomes, particularly regarding antibody titers, *Salmonella* reduction, and feed conversion ratio (FCR). Pooled analyses indicate that while overall probiotic supplementation may improve performance and selected immune markers, effect sizes vary considerably depending on strain identity, dosage, bird age, challenge model, and environmental conditions. In several meta-analyses, between-study heterogeneity (I²) was moderate to high, underscoring the inconsistency of responses across trials.

For example, *L. acidophilus* supplementation has frequently been associated with increased mucosal IgA secretion and, in some vaccination trials, elevated serum IgY titers; however, quantitative synthesis reveals that improvements in antibody response are not uniformly significant across all studies. Similarly, reductions in cecal *Salmonella* counts range from negligible to approximately 1 log₁₀ CFU/g depending on strain and experimental challenge conditions [41].

In contrast, *B. subtilis* strains tend to show more consistent improvements in growth performance parameters, particularly FCR, likely due to increased enzyme production and enhanced nutrient digestibility. Nevertheless, immune endpoints such as cytokine expression (e.g., Interferon-gamma [IFN-γ], interleukin [IL]-1β, IL-6, IL-10) and lymphoid organ indices (bursa, spleen, thymus weights) show variable modulation, suggesting that performance benefits do not always parallel measurable immune enhancement [42].

Multi-strain formulations (e.g., combinations of *Lactobacillus* spp. and *Bacillus* spp.) are sometimes reported to exert additive or synergistic effects on mucosal IgA levels and pathogen suppression. However, recent quantitative reviews caution that multi-strain products do not consistently outperform well-characterized single strains, and, in some analyses, the magnitude of the effect is comparable or highly context-dependent [43]. This finding reinforces the importance of precise strain-level evaluation rather than broad taxonomic categorization.

Taken together, these findings indicate that probiotic effects cannot be reliably inferred at the genus level. Table 2 summarizes the comparative effects of selected probiotic strategies in broiler chickens based on strain-specific evidence, highlighting differences in immune responses, lymphoid organ modulation, pathogen reduction, and production performance [41–43].

**Table 2:** Comparative summary of selected probiotic strategies in broiler chickens based on strain-specific evidence.

**Probiotic strategy**	**Immune response (IgA/** **IgY** **, cytokines)**	**Effects on lymphoid organs**	**Salmonella reduction**	**Performance (BWG/FCR)**	**Overall consistency of evidence**	**References**
*Lactobacillus acidophilus* (single strain)	Increased mucosal IgA with moderate consistency; serum IgY response variable across vaccination and challenge studies; cytokine modulation highly context-dependent	Mild increase in bursa weight reported in some trials; spleen and thymus responses inconsistent	Variable reduction, ranging from negligible effect to approximately 1 log₁₀ CFU/g in cecal content under challenge conditions	Modest improvement in FCR; BWG response generally variable	Moderate heterogeneity among studies; strain- and challenge-dependent outcomes	[41]
*Bacillus subtilis* (single strain)	Variable cytokine modulation (e.g., IFN-γ, IL-1β, IL-6, IL-10); limited consistent evidence for IgY enhancement	Inconsistent effects on bursa, spleen, and thymus weights	Strain-dependent and variable across experimental models	More consistent improvement in FCR and sometimes BWG, likely associated with enzyme production and improved nutrient digestibility	Relatively consistent for performance, but heterogeneous for immune endpoints	[42]
Multi-strain formulations	Potential additive or synergistic effects on mucosal IgA and cytokine responses; effect magnitude varies substantially	Variable and highly dependent on strain combination and dosage	Sometimes greater reduction than control groups; not consistently superior to optimized single strains	Variable effects on BWG and FCR; strongly context-dependent	High between-study heterogeneity; inconsistent superiority over single strain probiotics	[43]

## GENERAL MECHANISMS OF PROBIOTICS IN THE DIGESTIVE TRACT

Probiotics act through several interrelated biological mechanisms to maintain digestive health and support the immune system in poultry [52]. However, not all proposed mechanisms are supported by the same level of experimental evidence, and their consistency varies depending on strain, dosage, host age, and production conditions. Therefore, it is important to distinguish between well-established mechanisms and those that remain emerging or strain-dependent [12].

One important mechanism is competitive exclusion, the ability of probiotic microorganisms to compete with pathogens for colonization sites and nutrient sources on the intestinal epithelium [53]. Competitive exclusion against pathogens such as *Salmonella* spp., *E. coli*, and *Clostridium perfringens* is considered a well-established mechanism, particularly for *Lactobacillus* and *Bacillus* strains supported by *in vivo* challenge studies in broilers [20]. By occupying receptors typically used by pathogens, probiotics can inhibit the adhesion of harmful microorganisms such as *Salmonella* spp., *E. coli*, and *C. perfringens*; this mechanism is directly associated with reduced incidence of enteric infections and improved disease resistance in broilers and layers [54]. However, the magnitude of pathogen reduction is highly strain-specific and not uniformly reproducible across all experimental models or farm conditions. Furthermore, biofilm formation has been proposed as an additional protective mechanism, but evidence for stable biofilm-mediated protection in commercial poultry environments remains limited and should be regarded as an emerging hypothesis rather than a universally confirmed mechanism [55].

In addition to direct competition, probiotics also produce a variety of antimicrobial metabolites, including organic acids such as lactic, acetic, and butyric acids, as well as hydrogen peroxide and bacteriocins [56]. Organic acids help lower the pH of the intestinal lumen, creating less than ideal conditions for the growth of pathogenic bacteria [57]. Meanwhile, bacteriocins, antimicrobial peptides produced via ribosomal synthesis, provide an additional layer of protection by inhibiting pathogen cell wall formation or disrupting membrane integrity [58]. Nevertheless, bacteriocin-mediated pathogen inhibition is strongly strain-dependent, and *in vivo* efficacy does not always correlate with *in vitro* antimicrobial activity, highlighting inconsistency across studies. Thus, while antimicrobial metabolite production is biologically plausible and experimentally supported, its quantitative contribution to pathogen suppression under field conditions remains variable [59].

Probiotics help strengthen the integrity of the intestinal mucosa by modulating the expression of tight junction proteins, such as occludin, claudin, and zonula occludens [60]. Upregulation of tight junction gene expression has been repeatedly observed in controlled poultry experiments, particularly with selected *Lactobacillus* and *Bacillus* strains, suggesting that barrier reinforcement is a moderately well-established mechanism [61]. However, the translation of molecular changes into consistent improvements in growth performance or reduced systemic inflammation is not uniformly observed across all trials, indicating context dependency [62]. Increased mucus production by goblet cells has also been described, but evidence remains inconsistent, with some studies reporting significant effects while others show minimal or no change, suggesting strong strain and environmental interactions [63].

Another important mechanism is the ability of probiotics to alter the composition of the gut microbiota [64]. In commercial poultry production, probiotic supplementation has become increasingly prevalent, particularly following restrictions on AGPs, with industry reports indicating widespread adoption in broiler and layer operations across Europe and parts of Asia [8]. The presence of probiotic microbes helps establish a more stable microbial community, as reflected by an increase in beneficial bacteria such as *Lactobacillus* and *Bifidobacterium* and a decrease in the populations of pathogenic and opportunistic bacteria [65]. While shifts in microbial composition are commonly reported, the direction and magnitude of these changes vary substantially across studies, and causality between microbiota alteration and immune enhancement is not always clearly demonstrated. In many cases, microbiota modulation is inferred from correlation rather than mechanistic proof [66]. Moreover, some *Bacillus*-based products exert beneficial effects without persistent colonization, suggesting that transient metabolic or immunological signaling, rather than stable microbiota restructuring, may underlie observed benefits. This indicates that microbiota modulation is partly strain-dependent and not a universal requirement for probiotic efficacy [67].

Associations between probiotic supplementation and improved antibody titers following vaccination have been documented [21]. However, enhancement of post-vaccination immune responses appears highly strain-specific and influenced by timing, dosage, and baseline immune status, with some studies reporting significant improvements and others observing negligible effects [29]. Therefore, vaccine responsiveness should be interpreted as a promising but not universally guaranteed outcome of probiotic supplementation.

**Figure 1:**
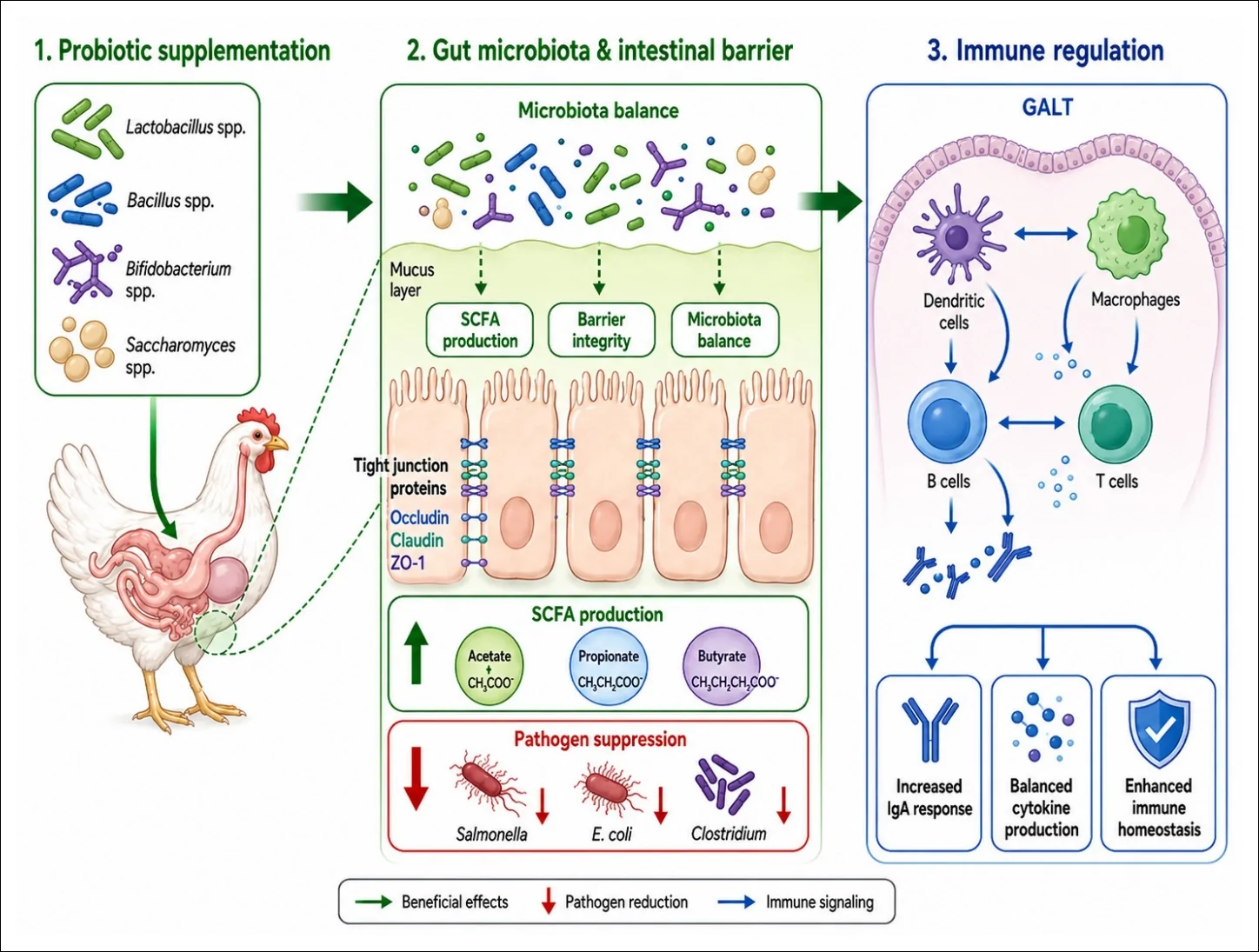
General mechanisms of probiotics in the digestive tract. This schematic illustration was conceptually developed based on published evidence [52–67] and generated using artificial intelligence tools (ChatGPT 5.2), then subsequently modified by the authors.

As shown in Figure 1[52–67], probiotics support poultry health through competitive exclusion, antimicrobial production, barrier modulation, and microbiota interaction. Among these, competitive exclusion and organic acid-mediated pathogen suppression are the most consistently supported mechanisms in poultry [53–59], whereas biofilm formation, extensive microbiota restructuring, and universal enhancement of vaccine responses remain emerging or strain-dependent phenomena requiring further controlled validation [55, 66, 67].

**POSTBIOTICS AND PARAPROBIOTICS:** NEXT-GENERATION ALTERNATIVES TO LIVE PROBIOTICS

Probiotics are traditionally defined as live microorganisms that confer health benefits to the host when administered in adequate amounts. However, recent advances in microbial biotechnology have expanded this concept beyond viable cells alone [13].

In addition to live probiotics, increasing attention has been directed toward postbiotics and paraprobiotics as next-generation functional feed additives. Paraprobiotics refer to inactivated (e.g., heat-killed) microbial cells that retain structural components capable of interacting with host immune receptors [68]. Postbiotics, by contrast, consist of microbial-derived metabolites or cell-free supernatants, including short-chain fatty acids (SCFAs), bacteriocins, peptides, teichoic acids, exopolysaccharides, and other bioactive compounds [69].

Recent meta-analyses (2024–2025) in broiler chickens indicate that postbiotics can significantly improve growth performance, FCR, villus height-to-crypt depth ratio, and both systemic and mucosal immune parameters. Several studies report enhanced intestinal morphology, increased expression of tight junction proteins, and modulation of cytokine profiles comparable to, or in some cases exceeding, the effects observed with live probiotic supplementation [15, 70, 71].

Mechanistically, live probiotics exert their effects through competitive exclusion, modulation of microbiota composition, metabolite production, and direct interaction with intestinal epithelial and immune cells [14]. Postbiotics and paraprobiotics, however, may act primarily through bioactive molecular signaling rather than colonization. Structural components such as peptidoglycan and lipoteichoic acid can interact with pattern recognition receptors (PRRs; e.g., TLRs), thereby stimulating controlled immune responses [72]. SCFAs and other metabolites contribute to epithelial integrity, anti-inflammatory signaling, and pathogen suppression. Because these preparations do not require microbial viability, their functional effects are less dependent on successful gut colonization [73].

From a commercial perspective, postbiotics and paraprobiotics offer several practical advantages. They demonstrate greater stability during feed pelleting and storage under high temperature and humidity, reducing losses in activity associated with heat-sensitive live cells [74]. Moreover, the absence of viable microorganisms minimizes concerns regarding horizontal gene transfer of ARGs, translocation, or unintended ecological persistence. These characteristics make them particularly attractive for intensive production systems where feed processing conditions may compromise live probiotic viability [75].

Nevertheless, certain limitations remain. Unlike live probiotics, postbiotics do not replicate or dynamically adapt to the gut environment, and their effects may depend on continuous supplementation [76]. Additionally, optimal dosing, standardization of bioactive components, and harmonized regulatory classification (as a feed additive vs. a functional metabolite) require further clarification [77].

## IMMUNE SYSTEM IN POULTRY

The avian immune system is a complex network of defense mechanisms involving the coordinated action of cellular and humoral components to protect the host against a wide range of infectious agents [78]. This defense system encompasses mucosal immunity, innate immunity, and adaptive immunity, all of which function in an integrated manner and are strongly influenced by the gut microbiota through the gut–immune axis [79]. A comprehensive understanding of the structure and function of this immune system is essential for evaluating the effectiveness of nutritional interventions, including probiotics, in improving poultry health and disease resistance [80].

## MUCOSAL IMMUNITY (GALT)

Mucosal immunity in birds, collectively referred to as GALT, is a crucial component of the avian defense system and serves as the first line of defense against pathogens entering the gastrointestinal tract [81]. Probiotics interact directly with GALT by influencing antigen sampling in Peyer’s patches and cecal tonsils, where microbial-associated molecular patterns (MAMPs) derived from probiotic strains are recognized by PRRs expressed on epithelial and immune cells. Through these interactions, GALT helps maintain immune homeostasis by integrating signals from microbes, nutrients, and environmental stimuli to generate effective yet controlled immune responses [82]. Rather than describing GALT solely as a structural immune compartment, this section emphasizes its functional role in mediating probiotic-driven immunomodulation, particularly through MAMP–PRR interactions that influence antigen presentation and stimulate IgA production in Peyer’s patches and cecal tonsils [83]. A summary of the principal components of the avian immune system, including GALT, innate immunity, and adaptive immunity, together with their main structures or cells and corresponding functions, is presented in Table 3[84–135].

Peyer’s patches, scattered throughout the small intestine, act as immune surveillance centers, involving M cells and various immune cells, including B lymphocytes, T lymphocytes, and dendritic cells [84]. Probiotic bacteria can be translocated by M cells to underlying dendritic cells, promoting antigen presentation and stimulating B cell differentiation into IgA-secreting plasma cells. Enhanced mucosal IgA production is one of the most consistently reported immune outcomes of *Lactobacillus* and selected *Bacillus* strains in poultry. IgA limits pathogen adhesion and neutralizes toxins at the epithelial surface [85]. IgA provides local protection by neutralizing pathogens and preventing microorganisms from adhering to the intestinal epithelium [86].

The bursa of Fabricius, a unique lymphoid organ in birds, serves as the primary site of B cell maturation and differentiation [87]. This organ not only plays a role in the production of diverse antibodies but also interacts with various antigens originating from the gastrointestinal tract, thereby supporting the emergence of humoral immune responses at both systemic and mucosal levels [88]. The role of the bursa of Fabricius is crucial, especially in the early stages of a bird’s life when the immune system is still developing [89].

The pharyngeal and cecal tonsils serve as antigen detection centers in the proximal and distal parts of the digestive tract [90]. The cecal tonsils, located at the base of the cecum, are one of the largest lymphoid tissues in birds and play a crucial role in processing signals from gut microbes [91]. Probiotic-mediated modulation of microbiota composition influences cytokine production within these tissues, promoting balanced Th1/Th2 responses and supporting vaccine-induced immunity. These structures are populated by T and B lymphocytes and antigen-presenting cells, which play a role in regulating inflammatory responses and maintaining immune tolerance to commensal microbiota [92].

## INNATE IMMUNITY

Innate immunity in birds serves as the initial defense against various infectious agents and plays a crucial role in maintaining physiological stability [93]. Various immune cells, such as macrophages, heterophils, dendritic cells, and natural killer (NK) cells, work together to recognize, phagocytose, and eliminate pathogens through rapid and nonspecific mechanisms. In recent years, probiotics, such as *Lactobacillus* spp., *Bifidobacterium* spp., and *Bacillus* spp., have been shown to enhance innate immune responses through both direct and indirect pathways [94]. However, these immunomodulatory effects are strain-specific and should not be generalized across all probiotic species or formulations.

**Table 3:** Components and functions of the avian immune system and their relationship to the gut–immune axis.

**Immune system component**	**Specific structures/cells**	**Main immunological function**	**Role of probiotics in immune modulation**	**References**
**Mucosal immunity (GALT)**	Peyer’s patches	Luminal antigen surveillance, M cell-mediated antigen sampling, antigen presentation, and differentiation of B cells into IgA-secreting plasma cells	Enhance mucosal IgA production, promote dendritic cell-mediated antigen presentation, inhibit pathogen adhesion, and strengthen epithelial barrier integrity	[84–86]
	Bursa of Fabricius	Primary site of B cell maturation, differentiation, and antibody repertoire development in birds	Promote B cell maturation and enhance systemic and mucosal humoral immune responses	[87–89]
	Pharyngeal tonsils and cecal tonsils	Antigen recognition in the proximal and distal intestinal tract, regulation of inflammatory responses, and maintenance of immune tolerance to commensal microbiota	Enhance T- and B cell activation, modulate local cytokine responses, and improve vaccine-induced mucosal and systemic immunity	[90–92]
**Innate immunity**	Macrophages	Phagocytosis and production of inflammatory mediators, including IL-1β and TNF-α	Enhance phagocytic activity and early cytokine production through pattern recognition receptor-mediated signaling	[93–97]
	Heterophils	Rapid degranulation, respiratory burst, and nonspecific elimination of pathogens	Enhance degranulation and respiratory burst activity, although responses are strain-dependent	[98]
	Dendritic cells	Antigen presentation and activation of T and B lymphocytes	Increase expression of co-stimulatory molecules and enhance lymphocyte activation and adaptive immune responses	[99–100]
	Natural killer (NK) cells	Cytotoxic elimination of virus-infected and abnormal cells	Enhance cytotoxic activity through probiotic-derived metabolites and microbiota-mediated immunomodulatory signaling	[101–104]
**Adaptive immunity**	B cells (humoral immunity)	Production of IgA, IgM, and IgY antibodies and establishment of immunological memory	Promote B cell proliferation, increase mucosal IgA secretion, and enhance post-vaccination antibody responses (e.g., NDV and IBDV)	[105–108]
	T lymphocytes (cellular immunity)	CD4⁺ T cells coordinate immune responses and regulate cytokine production; CD8⁺ T cells eliminate infected cells	Promote T cell proliferation and maintain balanced cytokine responses, particularly IL-10 and IFN-γ	[109–116]
**Gut–immune axis**	Intestinal epithelium, gut microbiota, and GALT	Maintenance of immune homeostasis, epithelial barrier integrity, microbial tolerance, and coordinated responses to pathogens	Maintain microbiota balance, promote short-chain fatty acid production, reinforce epithelial barrier function, and modulate innate and adaptive immunity	[117–129]
**Gut–immune–climate axis (proposed concept)**	Gut microbiota, intestinal epithelium, immune cells, and environmental stressors	Integration of thermal stress, immune resilience, microbial homeostasis, and host adaptation under climate-related challenges	Heat-stable probiotics may preserve microbiota balance, maintain mucosal immunity, and reduce physiological stress biomarkers during heat stress	[130–135]

Direct effects occur via interaction of probiotic-associated molecular patterns (e.g., peptidoglycan, lipoteichoic acid) with host PRRs, such as Toll-like receptors (TLRs), expressed on epithelial cells and innate immune cells. The magnitude and direction of PRR-mediated signaling vary depending on the specific strain and its structural components. This interaction activates intracellular signaling pathways that modulate cytokine production and immune cell activation [95].

Macrophages play a key role in phagocytosis and in the production of inflammatory mediators [96]. Probiotic administration has been shown to enhance macrophage phagocytic capacity and increase early-stage cytokine expression, such as interleukin-1β (IL-1β) and tumor necrosis factor-alpha (TNF-α), primarily through PRR-mediated signaling [97]. However, these effects are strain-specific and dose-dependent, and not all probiotic candidates produce uniform activation across studies. Notably, these responses have been documented for selected strains under controlled experimental conditions and may differ with other strains or dosages. Heterophils may exhibit increased degranulation and respiratory burst activity following supplementation, although such effects are not uniformly observed across all probiotic candidates [98].

Indirect effects are mediated through modulation of gut microbiota composition and the production of microbial metabolites, such as SCFAs, which influence immune cell differentiation and inflammatory balance. Again, the extent of microbiota-driven immune modulation depends on the colonization ability and metabolic profile of the specific probiotic strain used [99]. Dendritic cells, which function to bridge innate and adaptive immunity, exhibit increased expression of costimulatory molecules and antigen-presenting capacity when the gut microbiota is modulated by probiotics. This activation contributes to optimal lymphocyte stimulation and a more effective adaptive immune response [100]. NK cell cytotoxic activity may also increase, partly driven by metabolite-mediated immunomodulatory signals rather than direct microbial contact alone [101].

## ADAPTIVE IMMUNITY

Adaptive immunity in birds is a specific defense system that develops after exposure to an antigen [102]. This mechanism comprises two main components: a humoral response involving B cells and antibody production, and a cellular response dependent on T lymphocyte activity [103]. These two mechanisms complement each other in specifically recognizing and neutralizing pathogens, while also forming an immunological memory that allows for a faster and more effective response upon subsequent exposure [104].

The humoral response is characterized by the production of various immunoglobulins (Ig), including IgA, IgM, and IgY (equivalent to IgG in mammals) [105]. IgA is the primary antibody on the intestinal mucosal surface and helps protect the epithelium by neutralizing pathogens and toxins, thereby preventing excessive colonization [106]. IgM appears earliest in the first stage of infection and functions to activate the complement system, while IgY provides long-term systemic protection against circulating pathogens [107]. In birds, the bursa of Fabricius is an essential lymphoid organ where B cells mature and differentiate, enabling the formation of a diverse and effective antibody repertoire [108].

The cellular response involves T lymphocytes, which include CD4⁺ (T helper) and CD8⁺ (T cytotoxic) subsets [109]. CD4⁺ cells regulate the activity of various immune cells through cytokine production, while CD8⁺ cells are responsible for recognizing and destroying virus-infected or abnormal cells [110]. The activity of these two types of T cells is influenced by signals from dendritic cells and macrophages, which present antigens [111]. This collaboration ensures the formation of a specific and targeted adaptive immune response. Nevertheless, cytokine modulation does not consistently translate into measurable improvements in protective immunity, indicating that immunological biomarkers and functional protection are not always directly correlated [112].

Probiotic supplementation has been shown to enhance the adaptive immune response in poultry. However, these immunomodulatory effects are highly dependent on the specific probiotic strain, administered dose, duration of supplementation, and the type of vaccine used. These microorganisms can increase B and T cell proliferation, promote mucosal IgA secretion, and increase post-vaccination antibody titers, including against Newcastle disease virus (NDV) and Infectious Bursal Disease Virus (IBDV) [113]. Nevertheless, variations in probiotic dosage and vaccination protocols may lead to differing magnitudes, or even absence, of immune enhancement. Probiotic supplementation has been associated with increased mucosal IgA and, in some cases, enhanced post-vaccination antibody titers [114]. However, enhancement of vaccine responses is not universally observed and appears highly strain-, dose-, and context-dependent.

Nevertheless, several studies have reported inconsistent or limited effects of probiotics on vaccine-induced antibody titers, with some trials showing no significant improvement in NDV or IBDV seroconversion compared with non-supplemented controls (additional references to be included) [115]. In some cases, immune enhancement was transient, marginal, or statistically non-significant, particularly when baseline immune status was already optimal or when suboptimal probiotic doses were used.

Furthermore, probiotics influence cytokine regulation, including increases in IL-10 and IFN-γ, which help balance pro-inflammatory and anti-inflammatory immune responses [116]. However, cytokine modulation does not always translate into measurable improvements in vaccine efficacy, highlighting the complexity of host–microbe–vaccine interactions. These cytokine-modulating effects are context-dependent and may vary according to host genetics, immune status, and vaccine challenge conditions [116]. These effects not only increase resistance to infection but also reduce the risk of tissue damage caused by excessive inflammation.

## GUT–IMMUNE AXIS RELATIONSHIP IN POULTRY

The gut–immune axis is presented here as a functional framework describing probiotic interaction with intestinal epithelium, microbiota, and GALT, rather than as a generalized theoretical concept. The poultry digestive tract not only functions in digestion and nutrient absorption, but also plays a key role in immune system regulation through the gut–immune axis [117]. This concept describes the dynamic relationship between the gut microbiota, intestinal epithelial cells, and GALT, which collectively maintain immune stability and regulate responses to pathogens [118]. Through this system, poultry are able to balance tolerance to commensal microbes while activating defense mechanisms when encountering pathogenic microorganisms [119].

However, it is important to acknowledge that several detailed signaling pathways commonly cited in gut–immune discussions are extrapolated from mammalian models, while avian-specific molecular validation remains relatively limited. Thus, although functional outcomes, such as improved IgA production, balanced cytokine expression, and reduced pathogen load, are supported by poultry studies, mechanistic depth at receptor and transcriptomic levels in birds is still evolving [120].

The gut microbiota plays a key role as a link in the gut–immune axis. A balanced microbial community is capable of producing various bioactive metabolites, including SCFAs, vitamins, and antimicrobial peptides that serve as signals for the immune system [121]. SCFAs, for example, play a role in promoting the differentiation of regulatory T lymphocytes (Tregs), reducing the production of pro-inflammatory cytokines, and increasing the expression of tight junction proteins in the intestinal epithelium [122]. However, the majority of mechanistic data regarding SCFA-mediated immune modulation has been characterized in mammalian models, with fewer direct functional studies available in poultry. In particular, molecular characterization of SCFA receptors, downstream transcription factors, and epigenetic regulation in avian immune cells is still limited. In this way, the microbiota helps maintain the strength of the mucosal barrier and influences the regulation of both innate and adaptive immune responses [123].

The intestinal epithelium serves as both a physical barrier and a crucial point of communication between the microbiota and the immune system [124]. Epithelial cells produce mucus, antimicrobial peptides, and immune signaling molecules that can activate dendritic cells and macrophages [125]. Dendritic cells that capture antigens from the intestinal lumen then guide the differentiation of T and B lymphocytes within the GALT, thus promoting the formation of mucosal antibodies and activating effector T cells. Again, many of these epithelial–immune interaction pathways have been extensively described in mammals, and equivalent avian mechanisms are still under active investigation [126]. Comprehensive transcriptomic, proteomic, and receptor-level analyses in poultry remain relatively underrepresented compared with mammalian research.

The GALT is a key structure that carries out immune responses at mucosal surfaces. Probiotics modulate GALT activity by enhancing dendritic cell maturation, increasing expression of co-stimulatory molecules, and promoting regulatory T cell (Treg) differentiation, thereby strengthening immune tolerance while maintaining effective pathogen defense [127]. This lymphoid tissue, including Peyer’s patches, bursa of Fabricius, pharyngeal tonsils, and cecal tonsils, facilitates recognition of antigens from the lumen, stimulation of B cells to produce IgA, and coordination of T lymphocyte activity in regulating local and systemic immune responses [128]. GALT activity is also influenced by metabolites and microbiota components, including probiotics, which can enhance specific immune responses without causing excessive inflammation. Nevertheless, direct mechanistic confirmation of several probiotic–GALT interactions in poultry remains less comprehensive compared with mammalian systems. Thus, while functional outcomes have been observed, detailed molecular validation in avian species is still evolving [129].

**Figure 2:**
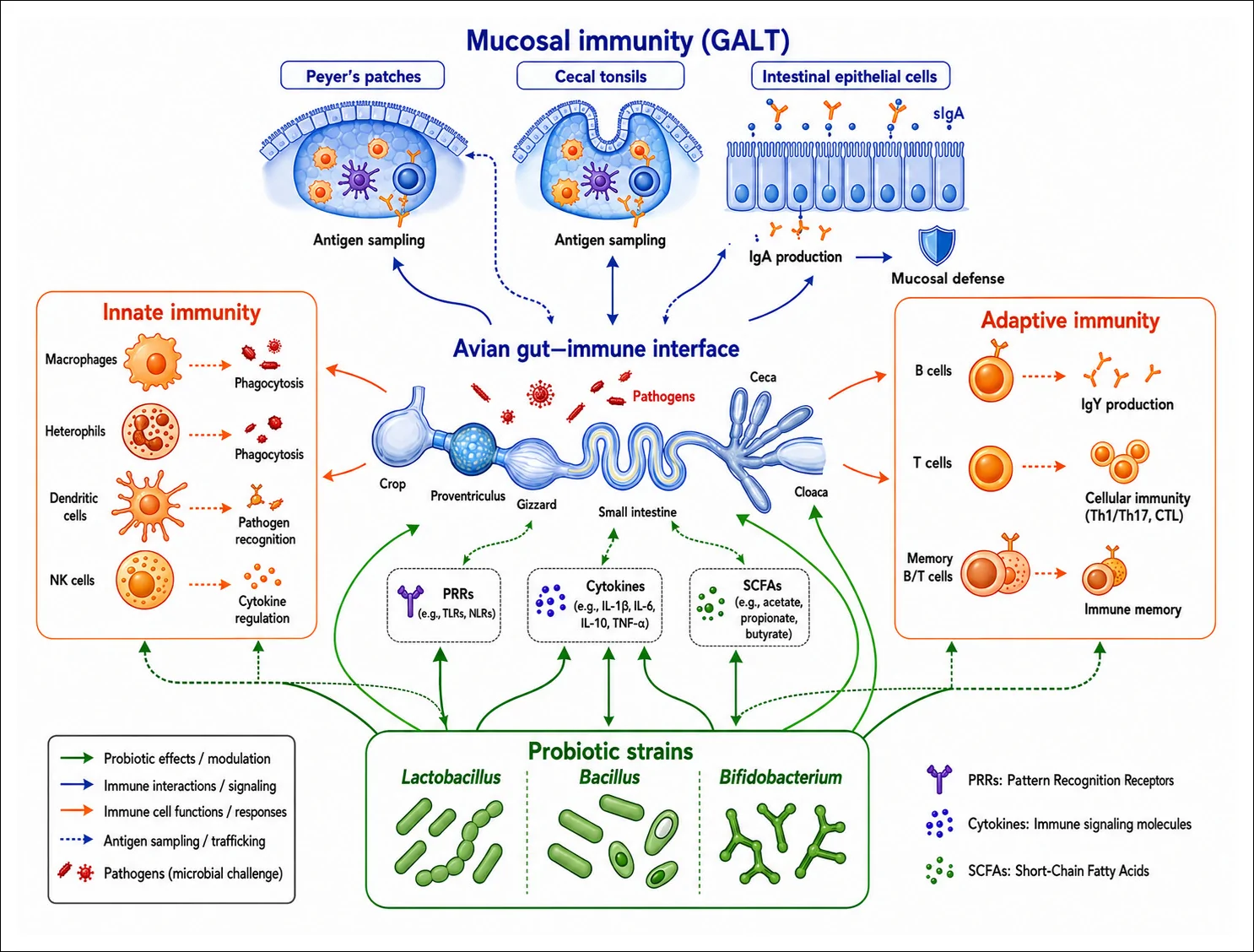
Innate and adaptive immunity in poultry, illustrating the gut–immune axis and proposed gut–immune–climate axis. This schematic illustration was conceptually developed based on published evidence [84–135] and generated using artificial intelligence tools (ChatGPT 5.2), then subsequently modified by the authors.

Harmonious interactions within the gut–immune axis play a crucial role in maintaining resistance to pathogens, controlling inflammatory responses, and supporting optimal performance in poultry. Through targeted interaction with GALT, probiotics enhance mucosal antibody production, improve post-vaccination antibody titers, and contribute to controlled inflammatory responses that favor growth efficiency in poultry [130]. However, given the current reliance on extrapolated mammalian data, further avian-specific mechanistic studies are needed to validate these proposed pathways. An imbalance in any one element, for example, microbial dysbiosis or damage to the intestinal epithelium, can trigger an excessive immune response, prolonged inflammation, or reduced growth efficiency [131]. Therefore, various nutritional and management approaches, including the administration of probiotics, prebiotics, and synbiotics, aim to maintain the stability of the gut–immune axis, strengthen intestinal barrier function, and regulate innate and adaptive immune activity [132]. As shown in Figure 2[84–135], the poultry immune system involves coordinated interactions between innate and adaptive immune components leading to antibody production. Furthermore, the figure highlights the dynamic interplay between gut microbiota, intestinal epithelium, and GALT, as well as the proposed gut–immune–climate axis under heat stress conditions [117–129, 133–135].

Furthermore, we propose extending this concept to a “Gut–Immune–Climate Axis” to emphasize the role of probiotics in climate resilience. Recent studies from 2025 indicate that heat stress and high ambient temperatures disrupt microbial balance, impair epithelial integrity, and suppress immune function in poultry [133]. Heat-stable probiotic strains, nanoparticle-encapsulated formulations, or multi-strain blends may mitigate these effects by preserving gut microbiota stability, enhancing mucosal immunity, and reducing stress biomarkers such as heterophil-to-lymphocyte ratio and corticosterone [134]. Integrating environmental and management factors, such as stocking density and thermal load, within this axis provides a framework for linking gut–immune modulation to climate-adaptive poultry production, aligning with broader sustainability goals [135]. Future research should test these predictions under controlled heat stress conditions and in commercial settings to quantify probiotic-mediated resilience.

## THE EFFECT OF PROBIOTICS ON POULTRY IMMUNITY

The application of probiotics in poultry nutrition is now a key approach to strengthening health and increasing resistance to various diseases. In addition to helping maintain the balance of the gut microbiota, probiotics can also influence the immune system, both innate and adaptive, thus enhancing the body's ability to fight pathogens and supporting successful vaccination [136]. However, immune activation is energetically demanding, and excessive or chronic immune stimulation may divert nutrients away from growth and production, potentially impairing feed efficiency and overall performance. Therefore, the goal of probiotic supplementation is to achieve balanced immunomodulation rather than maximal immune activation. A comprehensive understanding of how probiotics modulate immunity is an essential foundation for developing sustainable poultry nutrition strategies.

## PROBIOTICS IN INCREASING INNATE IMMUNITY

Innate immunity in birds serves as the initial defense against infection and involves various phagocytic cells, including macrophages, heterophils, and antigen-presenting cells such as dendritic cells. These components play a crucial role in recognizing and inhibiting pathogen development before the adaptive immune system kicks in [137]. Macrophage activation is a key process in the innate response, enhancing phagocytic capacity and the production of early inflammatory mediators [138]. Table 4 summarizes the effects of probiotics on the avian immune system, including innate and adaptive immune responses, lymphoid organ function, mechanisms for reducing stress and inflammation, and changes in gut microbiota composition [138–200].

**Table 4:** Effects of probiotics on poultry immunity, stress regulation, and gut microbiota.

**Immune domain**	**Components/organs**	**Main probiotic mechanisms**	**Functional impact on poultry**	**Key considerations/limitations**	**References**
**Innate immunity**	Macrophages	Enhance phagocytosis, stimulate early inflammatory mediator production, and activate NF-κB signaling through pattern recognition receptor-mediated pathways	Improve pathogen recognition, phagocytic efficiency, and early innate immune responses	Responses are strain-, dose-, and challenge-dependent; excessive activation may increase metabolic demands and oxidative stress	[138, 139]
	Heterophils	Enhance degranulation and respiratory burst activity	Improve rapid elimination of bacterial and opportunistic pathogens	Responses vary among probiotic strains and environmental conditions	[140]
	Dendritic cells	Upregulate co-stimulatory molecules and enhance antigen presentation	Promote activation of T and B lymphocytes and initiation of adaptive immune responses	Effects are generally more pronounced under pathogen challenge than under low-pathogen conditions	[141, 142]
	Natural killer (NK) cells	Enhance cytotoxic signaling and metabolite-mediated activation	Increase elimination of virus-infected and abnormal cells	Functional evidence in poultry remains relatively limited	[143–145]
**Adaptive immunity**	B cells/plasma cells	Promote proliferation and differentiation into IgA- and IgY-producing plasma cells	Enhance mucosal and systemic immunity and improve post-vaccination antibody responses (e.g., NDV, IBDV, and AI)	Responses vary according to production type, vaccination protocol, and probiotic strain	[146–155]
	T lymphocytes (CD4⁺ and CD8⁺)	Promote proliferation, activation, and cytokine-mediated immune coordination	Improve coordination of immune responses and elimination of infected cells	Increased T cell numbers do not always correspond to enhanced protective immunity	[156–158]
	Immunomodulatory cytokines	Modulate expression of IL-10, IFN-γ, IL-1β, IL-6, and TNF-α	Maintain a balanced pro- and anti-inflammatory immune response	Benefits are context-dependent, and excessive cytokine expression may be detrimental	[159–165]
**Major immune organs**	Bursa of Fabricius	Increase follicular diameter, B cell proliferation, and relative organ weight	Promote B cell maturation and enhance humoral immune competence	Morphological changes should be interpreted together with functional immune indicators	[166–176]
	Thymus	Increase thymocyte proliferation and cortex-to-medulla ratio	Promote T cell maturation and strengthen cellular immunity	Histological changes do not always indicate improved immune protection	[177]
	Spleen	Increase lymphoid cell density in the white pulp and enhance B- and T cell interactions	Strengthen systemic immune activation and antibody production	Increased organ weight alone may reflect transient immune activation rather than functional improvement	[178, 179]
**Stress and inflammation**	Heterophil-to-lymphocyte ratio and corticosterone	Reduce physiological stress indicators and inflammatory signaling	Decrease physiological stress and excessive inflammatory responses	Benefits are more evident under heat stress, high stocking density, or infectious challenge	[180–184]
	Reactive oxygen species and oxidative stress markers	Increase antioxidant enzyme activity (superoxide dismutase (SOD), GPx, and catalase) and reduce malondialdehyde and reactive oxygen species levels	Reduce oxidative damage and improve physiological resilience	Antioxidant effects depend on baseline oxidative stress and management conditions	[185–190]
**Gut microbiota and gut–immune axis**	Gut microbiota composition and microbial metabolites	Increase beneficial bacteria (Lactobacillus and Bifidobacterium), promote competitive exclusion of pathogens, and enhance short-chain fatty acid production	Strengthen epithelial barrier function, maintain immune homeostasis, and reduce pathogen colonization	Changes in microbial composition do not always correlate directly with measurable improvements in immune function	[191–200]

Probiotic supplementation with *Lactobacillus* spp., *Bifidobacterium* spp., and *Bacillus* spp. has been shown to stimulate macrophage activation through interactions with receptors on the surface of immune cells. This activation initiates immune signaling pathways, including nuclear factor kappa B (NF-κB), which enhances macrophages’ ability to ingest and destroy pathogens [139]. Nevertheless, excessive activation of inflammatory signaling pathways may increase metabolic costs and oxidative stress, which can negatively affect growth performance if not properly regulated. However, much of the mechanistic evidence for macrophage activation originates from *in vitro* cell culture studies or controlled experimental challenge models. In commercial *in vivo* settings, improvements in phagocytic activity or cytokine expression are sometimes modest or statistically non-significant, particularly in flocks with low pathogen pressure [140]. Furthermore, probiotics also enhance phagocytic activity, making immune cells more efficient in eliminating bacteria, viruses, and opportunistic microbes that invade the poultry digestive tract.

In addition to enhancing immune cell activity, probiotics regulate early-phase cytokine expression [141]. Research shows that probiotic supplementation can stimulate the production of pro-inflammatory cytokines, such as IL-1β, IL-6, and TNF-α, which function to coordinate the early immune response, attract additional immune cells, and strengthen mucosal defenses without causing chronic inflammation [142]. However, the induction of these cytokines is not universally beneficial; excessive or prolonged upregulation may contribute to tissue damage or chronic inflammation, depending on the host condition, pathogen challenge, and probiotic strain used. Notably, cytokine modulation appears to be strain-dependent, and dose–response relationships are not always linear. Several trials have reported minimal changes in innate immune gene expression with low probiotic doses, whereas excessively high doses did not proportionally enhance immune parameters and, in some cases, increased inflammatory markers without performance benefits [143].

## PROBIOTICS AND ADAPTIVE IMMUNITY

Adaptive immunity in birds is a specific defense system that develops after exposure to an antigen, involving humoral and cellular responses [144]. The humoral response is characterized by antibody production by B cells, while the cellular response is driven by T lymphocytes, including CD4⁺ (T helper) and CD8⁺ (T cytotoxic). These two pathways work synergistically to enhance the bird’s ability to recognize and neutralize specific pathogens and to build immunological memory, providing long-term protection [145]. While improved adaptive immunity can enhance disease resistance, overstimulation of lymphocyte proliferation and sustained cytokine production may increase maintenance energy requirements, thereby potentially reducing growth rates or egg production under certain conditions.

Probiotic supplementation, such as *Lactobacillus* spp., *Bifidobacterium* spp., and *Bacillus* spp., has been shown to increase antibody titers against vaccines and pathogens in poultry, including NDV, IBDV, and Avian Influenza (AI). This suggests direct stimulation of B cell proliferation and differentiation into antibody-producing plasma cells, particularly IgA in the mucosa and IgY in the systemic circulation, thereby enhancing both local and systemic protection [146].

Nevertheless, responses differ substantially between broilers and layers. Broilers often show short-term increases in antibody titers during early growth phases, particularly when probiotics are administered before or at the time of primary vaccination. In contrast, layers, due to their longer production cycle, tend to exhibit more variable humoral responses, with some studies reporting stabilization rather than significant elevation of antibody titers [147].

Importantly, several *in vivo* studies have reported no significant improvement in NDV or IBDV antibody titers despite probiotic supplementation, with seroconversion levels comparable to non-supplemented controls [148, 149]. In some cases, increases were statistically significant but biologically marginal, without clear improvement in protection following challenge tests.

These inconsistent outcomes may be influenced by probiotic strain specificity, suboptimal dosage, duration of administration, vaccine type, environmental stressors, or the baseline immune status of the birds. In well-managed flocks with adequate nutrition and low pathogen pressure, additional probiotic supplementation may yield only marginal or statistically non-significant improvements in humoral responses [150].

Clear links to vaccination protocols have now been incorporated. Studies indicate that probiotic adminis-tration initiated 1–2 weeks prior to primary vaccination and continued through booster doses may enhance peak antibody titers, whereas supplementation started only after vaccination often results in limited or delayed effects [151]. Moreover, some trials reported enhanced early antibody response (e.g., at 7–14 days post-vaccination) without sustained differences at later time points, suggesting transient rather than durable immunomodulation [152].

Additionally, the immunological response to probiotic supplementation may differ between broilers and layers due to their distinct genetic selection goals and production physiology [153]. Broilers, which are selected for rapid growth and short production cycles, may exhibit more pronounced short-term improvements in antibody titers and growth-associated immune efficiency, but they may also be more sensitive to immune-related energy trade-offs [154]. In contrast, layers, characterized by longer production periods and sustained metabolic demands for egg production, may benefit more from long-term immune stabilization and balanced cytokine regulation rather than marked increases in antibody titers alone [155]. Consequently, probiotic strategies should consider production type (broiler vs. layer) to optimize both immune competence and performance outcomes.

In addition to enhancing B cell activity, probiotics also affect T lymphocyte populations, including increasing the number and activity of CD4⁺ and CD8⁺ cells [156]. CD4⁺ cells play a role in supporting B cell activation and coordinating the immune response, while CD8⁺ cells are responsible for targeting and destroying virus-infected or abnormal cells [157]. However, increases in CD4⁺ or CD8⁺ cell counts are not consistently associated with improved vaccine efficacy or disease resistance, highlighting that quantitative lymphocyte expansion does not always translate into functional immune superiority [158]. This modulation results in a more efficient cellular response to antigens while enhancing protection against pathogen infection.

Probiotics also influence the cytokine profile, which has immunomodulatory properties [159]. Studies have shown increased expression of IL-10, an anti-inflammatory cytokine that helps maintain a balanced immune response, and increased IFN-γ, which supports the activation of T cells and macrophages to eliminate pathogens [160]. Yet, contradictory findings exist: some studies report unchanged or even reduced IFN-γ expression following supplementation, particularly under non-challenge conditions [161]. This variability underscores the absence of a clear strain hierarchy and indicates that probiotic efficacy is context-dependent rather than universally positive. Nevertheless, shifts in cytokine expression should not be interpreted as inherently advanta-geous in all contexts [162]. For example, excessive IFN-γ production may intensify inflammatory responses, whereas elevated IL-10 levels could potentially dampen protective immunity if overexpressed [163].

Therefore, the immunological benefit of cytokine modulation depends on achieving an appropriate balance between pro-inflammatory and anti-inflammatory signals rather than simple upregulation of specific cytokines [164]. Taken together, current evidence indicates that probiotic effects on vaccine-induced humoral immunity are variable and context-dependent rather than universally positive [165]. This combined effect on cytokines, lymphocyte counts, and antibody production confirms that probiotics function as natural immunomodulators, enhancing the effectiveness and specificity of adaptive immunity in poultry [166]. Optimal poultry performance depends on maintaining immune competence without triggering unnecessary or prolonged immune activation.

## EFFECTS ON MAJOR IMMUNE ORGANS

Primary and secondary lymphoid organs in birds, such as the bursa of Fabricius, thymus, and spleen, play a crucial role in the formation and maturation of the immune system [167]. The bursa of Fabricius is a typical avian organ that serves as the site of B cell differentiation and maturation, while the thymus is responsible for the development of T lymphocytes [168]. The spleen, as a secondary lymphoid organ, plays a role in monitoring blood circulation, activating immune cells, and producing systemic antibodies [169].

Probiotic supplementation has been shown to influence the morphological development and relative weight of major lymphoid organs in poultry [170]. While some broiler trials report increased relative bursa or spleen weight following supplementation, others show no significant differences compared with controls [171]. Moreover, organ enlargement has not consistently correlated with improved antibody titers or enhanced protection following pathogen challenge. Studies have shown that administering probiotics, such as *Lactobacillus* spp., *Bifidobacterium* spp., and *Bacillus* spp., can increase the relative weight index of the bursa, thymus, and spleen, reflecting stimulation of immune cell proliferation and lymphoid tissue maturation [172]. This increase in relative weight is generally associated with increased lymphocyte counts and enlarged lymphoid follicles, indicating more immunologically active organs [173].

However, enlargement of lymphoid organs does not necessarily equate to improved immune competence. Increased organ weight may reflect transient immune activation, physiological adaptation, or even inflammatory responses rather than enhanced protective immunity [174]. Therefore, morphological enlargement should be interpreted alongside functional indicators, including antibody titers, cytokine profiles, pathogen challenge outcomes, and overall health performance, to accurately assess immunological benefits [175].

In addition to quantitative effects, probiotics also affect the histological structure of lymphoid organs [176]. The bursa of Fabricius showed increased lymphoid follicle diameter, cortical thickening, and a higher number of B cells, while the thymus experienced thymocyte proliferation and an increased cortex-to-medulla ratio [177]. In the spleen, probiotic supplementation increased lymphoid cell density in the white pulp and strengthened interactions between B and T cells [178]. These histological changes indicate that probiotics not only affect organ size but also enhance the functional ability of lymphoid organs to respond to antigens [179]. Nevertheless, sustained or excessive enlargement without corresponding improvements in immune efficiency may indicate immune overstimulation or increased metabolic burden, underscoring the need for balanced immune modulation rather than maximal organ growth.

## PROBIOTICS IN REDUCING STRESS AND INFLAMMATION

Physiological stress in poultry, which can arise from environmental factors, overcrowding, or infection, increases the production of reactive oxygen species (ROS) and activates inflammatory pathways such as NF-κB, leading to tissue damage and reduced performance [180]. NF-κB activation increases the expression of pro-inflammatory cytokines, including TNF-α, IL-1β, and IL-6, which further exacerbates inflammation and oxidative stress [181].

Probiotic supplementation, including *Lactobacillus* spp., *Bifidobacterium* spp., and *Bacillus* spp., has been shown to suppress NF-κB activation, thereby reducing pro-inflammatory cytokine production and preventing excessive inflammation in the gastrointestinal tract and systemic tissues [182]. Furthermore, probiotics can enhance endogenous antioxidant capacity by stimulating the expression of enzymes such as superoxide dismutase (SOD), glutathione peroxidase (GPx), and catalase, thereby reducing ROS levels and other markers of oxidative stress, including malondialdehyde (MDA) [183].

Nonetheless, reductions in inflammatory markers are not universally observed across all experimental conditions. In low-stress environments, probiotic supplementation sometimes results in negligible changes in H/L ratio or corticosterone levels, suggesting that benefits may be more pronounced under stress or pathogen challenge rather than under optimal management conditions [184].

The effects of probiotics on stress are also evident in physiological indicators, such as the heterophil/ lymphocyte (H/L) ratio and corticosterone levels [185]. Studies have shown that probiotic supplementation reduces the H/L ratio, a marker of chronic stress in poultry, and also reduces plasma corticosterone levels, a glucocorticoid hormone that increases in response to stress. Thus, probiotics not only reduce physiological stress and inflammation but also help maintain immune balance and support poultry performance [186].

## PROBIOTIC–MICROBIOTA INTERACTIONS IN SUPPORTING IMMUNITY

The gut microbiota of poultry plays a crucial role in maintaining immune homeostasis and gastrointestinal health. Microbiota imbalance (dysbiosis), which can occur due to stress, infection, or antibiotic use, is often associated with increased pathogen colonization and a decreased immune response [187]. Probiotic supplemen-tation helps modify the microbiota and restore balance, supporting innate and adaptive immune function [188].

It should be emphasized that microbiota modulation does not always translate directly into measurable immune enhancement. Some studies demonstrate significant shifts in microbial composition without parallel increases in antibody titers or cytokine responses, indicating that microbial changes alone are insufficient predictors of functional immunity [189, 190].

Recent advances in multi-omics technologies, including *16S rRNA* gene sequencing, shotgun metagenomics, metabolomics, and transcriptomics, have provided deeper mechanistic insights into probiotic–microbiota–host interactions [191]. High-throughput sequencing analyses frequently report increases in alpha diversity indices (e.g., Shannon and Chao1) following probiotic supplementation, although the magnitude and consistency of these changes vary among strains and production environments [192]. Beta-diversity analyses often reveal distinct clustering of treated versus control groups, suggesting measurable shifts in microbial community structure [193].

One of the primary mechanisms of action of probiotics is to restore the composition of the gut microbiota [194]. Probiotics can increase the number of beneficial bacteria, such as *Lactobacillus* spp. and *Bifidobacterium* spp., which play a role in producing bioactive metabolites, SCFAs, and antimicrobial peptides. These metabolites not only inhibit the growth of pathogens but also interact with epithelial cells and GALT to strengthen the integrity of the mucosal barrier and stimulate immune cell activity [195].

Metabolomic profiling has further linked probiotic-induced microbiota shifts to increased production of SCFAs, particularly acetate, propionate, and butyrate. SCFAs serve not only as energy substrates for enterocytes but also as signaling molecules that regulate tight junction protein expression, mucin production, and anti-inflammatory pathways [196]. Integrative analyses using multivariable association models (e.g., MaAsLin2) have identified significant associations between specific bacterial taxa and host immune markers, including correlations between butyrate-producing taxa and increased mucosal IgA levels or anti-inflammatory cytokine expression [197].

In addition to increasing the number of beneficial bacteria, probiotics also help reduce the population of pathogens such as *Salmonella* spp., *E. coli*, and *Clostridium perfringens*. This occurs through competition for space and nutrients (competitive exclusion), the production of antimicrobial compounds such as organic acids and bacteriocins, and an enhanced mucosal immune response that limits pathogen colonization [198]. Thus, probiotics function as mediators of microbial ecology, maintaining a balance between commensal and pathogenic microbiota. However, competitive exclusion effects are strain-specific, and not all probiotic formulations consistently reduce pathogen load *in vivo*. Variability in colonization ability and farm hygiene conditions further complicates the establishment of a definitive strain hierarchy [199].

At the host molecular level, transcriptomic studies demonstrate that probiotic supplementation can modulate key immune and stress-related pathways, including suppression of NF-κB activation under heat or pathogenic stress conditions, upregulation of tight junction–related genes (e.g., occludin, claudins), and modulation of cytokine gene expression (e.g., IFN-γ, IL-10, and IL-1β) [200]. Emerging evidence also suggests potential involvement in T regulatory (Treg) cell differentiation and epigenetic modulation through histone acetylation pathways influenced by SCFAs, although these mechanisms require further validation in poultry models [201].

**Figure 3:**
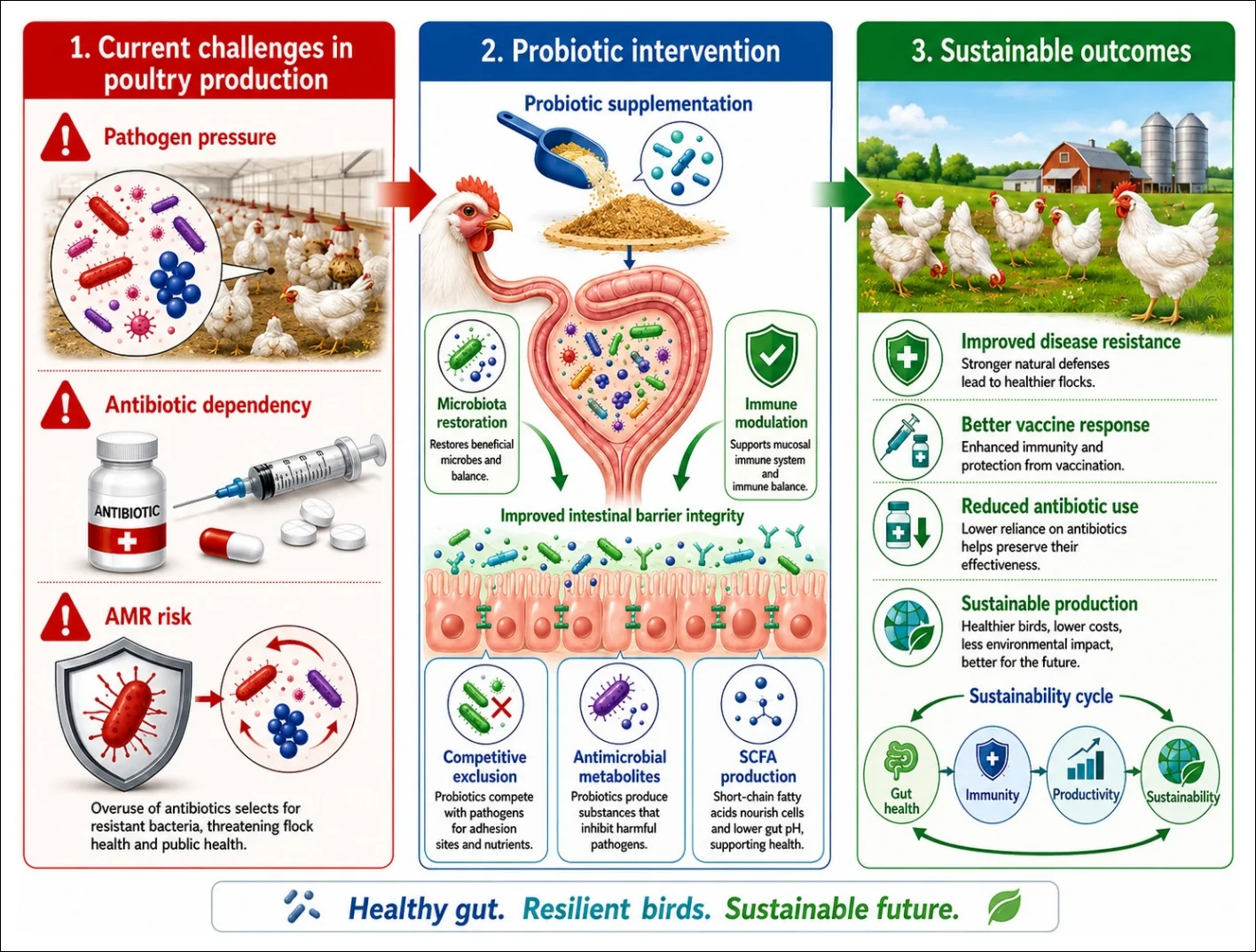
Gut–immune axis in poultry showing the bidirectional interaction between gut microbiota, intestinal epithelial barrier, and gut-associated lymphoid tissue (GALT). This schematic illustration was conceptually developed based on published evidence [191–200] and generated using artificial intelligence tools (ChatGPT 5.2), then subsequently modified by the authors.

The synergistic interaction among probiotics, the microbiota, and the immune system contributes to increased resistance to infection, strengthened mucosal immune response, and enhanced vaccine effectiveness and overall gastrointestinal health. Therefore, modulating the microbiota with probiotics is a crucial strategy in modern poultry production to naturally and sustainably improve performance, health, and pathogen resistance [202]. As illustrated in Figure 3[191–200], the gut–immune axis in poultry highlights the bidirectional interaction between gut microbiota, the intestinal epithelial barrier, and GALT, where microbial metabolites modulate immune responses while immune signaling maintains epithelial integrity and microbial balance.

## THE EFFECT OF PROBIOTICS ON DISEASE RESISTANCE IN POULTRY

Improving disease resistance is a key focus in modern poultry production, particularly in efforts to reduce antibiotic use [203]. Probiotics have been shown to play a crucial role in strengthening poultry resistance to viral and bacterial infections through two distinct but complementary mechanisms: immune priming and direct antimicrobial activity [16].

Resistance to viral infections is primarily mediated through enhancement of the host adaptive immune response rather than direct antiviral activity. In this context, probiotics primarily function as immune-priming agents, enhancing baseline immune readiness prior to pathogen exposure [22]. Several studies have shown that probiotic administration can increase the effectiveness of vaccines against major pathogens in poultry, such as NDV, IBDV, and AI [204–206]. This antiviral protection is associated with stimulation of mucosal and systemic immunity, increased production of virus-specific antibodies (mucosal IgA and circulating IgY), enhanced antigen presentation, and activation of CD4⁺ and CD8⁺ T lymphocytes, resulting in a faster and more robust immune response upon viral exposure [13]. Importantly, these effects are preventive rather than therapeutic, as probiotics do not directly inactivate viruses but instead enhance the host’s immunological preparedness prior to or during vaccination.

In contrast, resistance to bacterial infections involves both immune-mediated and direct microbiological mechanisms. Unlike immune priming against viruses, probiotic effects against bacteria often include direct antimicrobial actions within the gastrointestinal tract [37]. In addition to their effects on viral infections, probiotics are effective in reducing colonization by pathogenic bacteria such as *Salmonella* spp. and *Campylobacter* spp., which are common causes of enteric diseases and food contamination [207]. Unlike viral protection, which depends largely on adaptive immunity, antibacterial effects are strongly linked to competitive exclusion within the gastrointestinal tract. Probiotics suppress pathogen growth through competition for space and nutrients, production of antimicrobial metabolites such as organic acids and bacteriocins, and stimulation of mucosal immune responses that limit pathogen colonization in the gastrointestinal tract [208].

From a preventive perspective, continuous probiotic supplementation can reduce initial pathogen colonization and intestinal shedding [150]. From a therapeutic perspective, when administered during or after bacterial challenge, probiotics may help mitigate disease severity by restoring microbiota balance and modulating inflammatory responses; however, therapeutic effects are generally supportive rather than curative and do not replace antimicrobial treatment in severe infections [12].

The reduction of *Campylobacter* spp. colonization is particularly relevant for food safety. Lower intestinal loads and fecal shedding may translate into reduced carcass contamination at slaughter, thereby decreasing the risk of zoonotic transmission to humans [209]. However, reported reductions in *Campylobacter* counts vary widely among studies, and complete eradication is rarely achieved, highlighting the strain-specific and context-dependent nature of this effect [210].

It is important to note that the magnitude of probiotic effects may vary considerably under field conditions compared to controlled experimental settings. In commercial production systems, factors such as housing density, litter management, environmental stress (temperature and humidity fluctuations), feed formulation, water quality, vaccination programs, biosecurity standards, and existing gut microbiota composition can influence probiotic efficacy [211]. Consequently, responses observed in research trials may not always be directly replicated at the farm level. This field-level variability underscores the need for context-specific evaluation and optimization of probiotic strains, dosages, and administration strategies within different production environments [212].

The combined effects of immune priming, direct antimicrobial activity, and reduced pathogen shedding may positively impact poultry production performance, such as increased body weight, feed conversion efficiency, and meat and egg quality [3]. Thus, probiotics not only enhance poultry health but also naturally and sustainably enhance disease resistance, making them an effective nutritional strategy in modern poultry production [17].

## FACTORS THAT INFLUENCE THE SUCCESS OF PROBIOTICS

The success of probiotic administration to poultry is influenced by various factors that determine the viability, colonization ability, and immunomodulatory effectiveness of the microorganisms [213]. These factors include the dose and duration of administration, dosage form, resistance to pH and temperature of the digestive tract, use in combination with prebiotics (synbiotics), and the type of bacterial strain applied [214]. A thorough understanding of these aspects is crucial to ensure probiotics provide optimal benefits for poultry health, immune system function, and production performance [52].

In addition to biological factors, economic feasibility and regulatory compliance are critical determinants of successful probiotic implementation in commercial poultry systems. Therefore, probiotic selection should be based not only on scientific efficacy but also on cost–benefit considerations and regional regulatory frameworks [15].

To enhance the practical applicability of this section, a simplified decision-making framework for practitioners is proposed. Before probiotic implementation, producers should (1) define the primary objective (e.g., growth promotion, vaccine response enhancement, pathogen reduction, or gut health improvement); (2) select strains with documented efficacy for the intended goal and proven safety in poultry; (3) determine the appropriate dose and duration based on bird age, production phase, and farm conditions; (4) choose a dosage form compatible with feed processing and storage conditions; (5) consider synbiotic combinations when improved colonization or immune stimulation is required; and (6) monitor key performance and health indicators (body weight gain, FCR, mortality, morbidity, and antibody titers when available) to evaluate effectiveness and adjust strategies accordingly [37]. This structured approach enables evidence-based, farm-specific probiotic application under commercial conditions.

From an economic perspective, farmers and nutritionists should compare product cost per ton of feed with expected returns, such as improvements in FCR, reduced mortality, enhanced vaccine responsiveness, lower pathogen load, and potential premium value linked to antibiotic-free production [215]. Because probiotic responses are context-dependent, on-farm trials and performance benchmarking are recommended to verify economic return under specific production conditions [22].

## DOSAGE AND DURATION OF ADMINISTRATION

The effectiveness of probiotics in supporting poultry health and immunity depends heavily on the dosage and duration of administration [80]. Appropriate dosing is crucial for stabilizing beneficial microbial populations in the digestive tract, enabling them to compete with pathogens, produce bioactive metabolites, and effectively modulate the immune system [216]. Dosage is typically determined by the type of probiotic, the age of the poultry, environmental conditions, and the intended purpose, such as enhancing growth, strengthening mucosal immunity, or improving the response to vaccination [217].

The duration of probiotic administration also plays a significant role [218]. Short-term administration may be sufficient to trigger a temporary immune response, but long-term supplementation has been shown to be more effective in establishing a stable gut microbiota balance, increasing mucosal antibody (IgA) production, and strengthening innate and adaptive immunity [219]. Research shows that administering probiotics early in life in birds can stimulate the development of lymphoid organs, such as the bursa of Fabricius, thymus, and spleen, thus supporting optimal immune system maturation [220].

Furthermore, administering probiotics at too low a dose may have only a minimal effect, whereas administering too high a dose does not always confer benefits and can disrupt the microbiota balance [221]. From a cost–benefit standpoint, identifying the minimal effective dose is essential to avoid unnecessary product expenditure without proportional performance gains. Non-linear dose–response patterns suggest that increasing dosage beyond the optimal threshold may increase cost without improving biological outcomes [222]. This approach aims to ensure probiotics provide optimal benefits in improving health, disease resistance, and sustainable poultry production performance [223].

## DOSAGE FORM

The dosage form of a probiotic is a crucial factor in determining the stability, viability, and biological effectiveness of the microorganism when consumed by poultry [224]. Probiotics are available in various forms, such as liquid solutions, capsules, or freeze-dried, each with its own advantages regarding storage, resistance to digestive tract conditions, and ease of application in feed [225].

In commercial practice, the choice of dosage form should also consider economic efficiency, storage logistics, compatibility with feed mill infrastructure, and potential losses during pelleting [12]. Heat-stable spore-forming strains (e.g., *Bacillus* spp.) may reduce viability losses during feed processing, thereby improving cost-effectiveness compared to heat-sensitive strains requiring protective technologies [226].

In commercial poultry production, probiotic inclusion levels commonly range from 10⁶ to 10⁹ CFU/g of feed, which is equivalent to approximately 10⁷–10⁹ CFU/bird/day depending on feed intake and bird age [227]. Lower inclusion rates (10⁶ CFU/g) are typically used for maintenance of gut microbial balance, whereas higher levels (10⁸–10⁹ CFU/g) are often applied during early-life stages, periods of stress, vaccination, or pathogen challenge. However, optimal dosage remains strain-specific and should be validated under field conditions [222].

Liquid probiotics are generally suspensions of live bacteria in a culture medium, allowing them to be added directly to feed or drinking water. This format simplifies distribution and dosage adjustments, but has limitations related to shelf life and susceptibility to high temperatures and oxygen exposure [208].

Probiotics in capsule form provide physical protection against the acidic environment of the stomach while simplifying dosage adjustments. These capsules typically contain live bacterial cells coated in a protective matrix, increasing the likelihood that the bacteria will remain viable once they reach the intestines [228]. The capsule format also simplifies implementation in long-term supplementation programs.

Freeze-drying is the most commonly used preservation method for poultry. This process carefully removes water from the bacterial culture, keeping the cells viable in a dry, stable state for long-term storage [229]. Advantages of this method include resistance to high temperatures during feed mixing and a longer shelf life, while maintaining bacterial viability after rehydration in the digestive tract [230].

Recent advances highlight the use of nanotechnology and advanced delivery platforms to enhance probiotic stability, bioavailability, and immune effects [231]. Nanoparticle-based encapsulation protects probiotic cells from heat, oxygen, and stomach acidity, ensuring higher survival rates and improved colonization of the gut [232]. Such formulations have been shown in 2025 trials to improve villus height, enhance mucosal immunity, reduce mortality, and support growth performance under commercial conditions [233]. Water-delivered microencap-sulated probiotics allow precise dosing, rapid gut delivery, and minimal loss during feed processing.

Regarding duration of administration, short-term supplementation (7–14 days) is often applied around critical periods such as post-hatch, feed transition, vaccination, or disease challenge to enhance immune responsiveness [234]. In contrast, continuous supplementation throughout the production cycle (e.g., 4–6 weeks in broilers or extended periods in layers) has been associated with more stable gut microbiota establishment, improved feed efficiency, and sustained immune modulation [15]. Early-life administration, particularly during the first week post-hatch, appears especially important for promoting immune organ development and long-term microbiota stability [235].

Dose–response studies generally indicate a positive response up to an optimal threshold, beyond which benefits plateau and, in some cases, excessive doses may disturb microbial balance [131]. This suggests a non-linear dose–response relationship rather than a simple “more is better” effect. Therefore, probiotic application should consider both minimal effective dose and economic efficiency, ideally supported by controlled trials or farm level performance monitoring [80].

The choice of probiotic dosage form should be tailored to the intended use, the age of the birds, environmental conditions, and the method of administration [236]. Using the right dosage form ensures the viability of the probiotic bacteria, optimal immunomodulatory effects, and maximizes the benefits for digestive health and poultry performance [237].

## STABILITY AND RESISTANCE TO PH AND TEMPERATURE

The effectiveness of probiotics in supporting poultry health and immunity is largely determined by the stability and resistance of microorganisms to environmental conditions, particularly the pH of the digestive tract and the temperature during feed processing [22]. The poultry digestive tract exhibits significant pH variation, ranging from an acidic environment in the proventriculus (pH 2–4) to neutral or slightly alkaline conditions in the small intestine (pH 6–7.5) [238]. To remain viable until reaching the intestine, probiotics must be able to survive gastric acid, adhere to the epithelium, interact with the GALT, and modulate the poultry immune system [239].

Regulatory approval often requires documented stability, safety, and strain identification. In the European Union, probiotic strains used as feed additives must undergo formal safety and efficacy assessment before authorization, whereas regulatory frameworks in parts of Asia and developing regions may vary in stringency and enforcement [240]. Consequently, product quality, labeling accuracy, and viable cell counts may differ across markets, influencing both efficacy and producer confidence.

Besides pH, temperature tolerance is also a crucial factor, especially when probiotics are added to feed that is heated during pelletization (70–90°C) [241]. Probiotic microorganisms, including *Lactobacillus* spp., *Bifidobacterium* spp., and *Bacillus* spp., have varying heat tolerances [242]. Spore-forming bacteria, such as *Bacillus* spp., are more resistant to high temperatures than non-spore-forming bacteria [243]. Meanwhile, non-spore-forming probiotics typically require additional protection, such as encapsulation or a protective matrix, to maintain their viability during feed processing and storage [244].

Strategies for stabilizing probiotics include methods such as freeze-drying, microencapsulation, and the use of prebiotics as a protective matrix, allowing bacterial cells to remain viable during storage and passage through the digestive tract [245]. Probiotics that are resistant to gastric pH and feed processing temperatures have a greater chance of reaching the intestine in a viable state, establishing colonization, and providing optimal immunomodulatory effects, including stimulation of innate and adaptive immune cells, increased antibody production, and regulation of the gut microbiota [13]. Compliance with regional regulations regarding microbial safety, absence of transferable ARGs, and accurate strain declaration is increasingly important for international trade and consumer acceptance, particularly in antibiotic-reduction programs [202].

## COMBINATION WITH PREBIOTICS (SYNBIOTICS)

Synbiotics are a combination of probiotics and prebiotics designed to provide a synergistic effect in supporting gut health and the immune system of poultry [246]. Probiotics provide live beneficial microorganisms, while prebiotics, typically in the form of non-digestible oligosaccharides, serve as a specific nutrient source for the growth and activity of beneficial microbes [247]. This combination enhances probiotic colonization in the gastrointestinal tract, stimulates the production of bioactive metabolites, and modulates both innate and adaptive immune responses [248]. From a practical and economic perspective, synbiotics may offer improved colonization and immune outcomes; however, their higher formulation cost must be justified by measurable improvements in performance, health status, or pathogen reduction under field conditions [249].

Administering synbiotics to poultry has been shown to strengthen the integrity of the intestinal epithelium by increasing mucosal thickness, villus length, and the villus/crypt ratio [250]. This promotes more efficient nutrient absorption and strengthens physical defenses against pathogens [251]. Immunologically, synbiotics stimulate phagocyte activity, increase T- and B-lymphocyte proliferation, and promote the production of mucosal (IgA) and systemic (IgY) antibodies, resulting in a more effective immune response to pathogens and vaccines [252].

Furthermore, synbiotics have been shown to be more effective than single probiotics in suppressing the colonization of pathogens, such as *Salmonella* spp. and *Clostridium perfringens*, through mechanisms such as competition for space and nutrients, production of antimicrobial metabolites, and stimulation of local immune responses [253]. Restoring this microbiota balance also helps reduce intestinal inflammation and oxidative stress, thus supporting improved poultry performance, including body weight gain and feed conversion efficiency [254].

## BACTERIAL STRAINS USED

The selection of bacterial strains is a crucial factor in determining the effectiveness of probiotics in poultry, as each strain has unique capabilities in gut colonization, immune system modulation, and pathogen growth inhibition [255]. Commonly used strains include *Lactobacillus* spp., *Bifidobacterium* spp., *Bacillus* spp., *Enterococcus* spp., and *Streptococcus* spp., each with its own distinct metabolic characteristics and immunomo-dulatory effects [28].

*Lactobacillus* spp. are among the most commonly used probiotic strains in poultry [40]. This bacterium can colonize the intestinal epithelium, producing lactic acid to lower the lumen's pH and inhibiting the growth of enteric pathogens such as *Salmonella* spp. and *E. coli* [256]. Furthermore, *Lactobacillus* can stimulate the phagocytic activity of macrophages and heterophils and increase antibody production at both the mucosal and systemic levels [97].

*Bifidobacterium* spp. functions in the fermentation of oligosaccharides into SCFAs, which help maintain the integrity of the intestinal epithelium and act as immune signals for dendritic cells and lymphocytes [257]. These metabolites promote immune cell proliferation, reduce excessive inflammation, and strengthen the adaptive immune response to vaccines and pathogen infections [258].

*Bacillus* spp., particularly spore strains, are highly resistant to heat and stomach acid, allowing them to survive in the intestines [259]. Furthermore, *Bacillus* spp. can produce digestive enzymes, bacteriocins, and antimicrobial metabolites that support the growth of beneficial microbiota while suppressing pathogen colonization [260].

*Enterococcus* spp. and *Streptococcus* spp. are used as probiotics for their ability to stimulate both mucosal and systemic immune responses and to compete with pathogens for space and nutrients [261, 262]. These strains are often combined with *Lactobacillus* or *Bacillus* to enhance their synergistic effects in modulating the immune system and supporting gastrointestinal health [263].

Beyond biological performance, strain selection should consider regulatory approval status in the target market, documented safety (including absence of virulence factors or transferable resistance genes), and consistency of commercial production. For multinational poultry operations, differences in approval status across regions (e.g., EU vs. various Asian or other developing countries) may influence product availability and formulation strategies [264].

The selection of probiotic strains requires consideration of their resistance to the poultry digestive tract, colonization capacity, immunomodulatory potential, and safety [265]. Ultimately, successful probiotic implementation requires integration of biological efficacy, regulatory compliance, and economic return [15]. A structured evaluation combining scientific evidence, farm level performance data, and cost analysis provides a rational framework for decision-making in modern poultry production systems. A suitable combination of strains can enhance the effectiveness of probiotics in strengthening innate and adaptive immunity, increasing resistance to infection, and supporting sustainable poultry production performance [17].

## IN OVO AND EARLY-LIFE PROBIOTIC ADMINISTRATION

Early probiotic administration has been recognized as a strategy to optimize the establishment of the gut microbiota and immune development in poultry. Recent advances (2024–2025) have substantially expanded the evidence base for *in ovo* probiotic delivery, particularly injection at embryonic day 18 (ED18) into the amniotic cavity [235, 266]. Several studies using *Lactobacillus*-based single strains or multi-strain cocktails have demonstrated improved hatchability, enhanced early chick viability, and reduced colonization by opportunistic bacteria, including *Klebsiella* spp. and *Enterococcus* spp. during the first week post-hatch [267, 268]. These findings suggest that microbiota modulation can begin prior to hatch, during a critical window of immune ontogeny.

Mechanistically, *in ovo* administration has been associated with modulation of local immune responses in GALTs, particularly the cecal tonsils [269]. Reported effects include altered expression of pro- and anti-inflammatory cytokines such as IFN-γ, IL-1β, and IL-8, indicating early immune priming [270]. While some cytokine modulation appears transient during the immediate post-hatch phase, emerging evidence suggests that early microbial exposure may shape longer-term immune responsiveness and mucosal barrier function [114].

Comparative studies indicate that *in ovo* delivery can influence early microbial succession patterns in a manner comparable to repeated oral dosing post-hatch. Chicks receiving *in ovo* *Lactobacillus* cocktails often show accelerated establishment of beneficial lactic acid bacteria and reduced relative abundance of Enterobacteriaceae during the first two weeks of life [271]. In some trials, these early shifts were associated with sustained improvements in feed efficiency, body weight gain, gut morphology (e.g., villus height), and reduced pathogen load later in the production cycle [272].

Despite these promising findings, several factors influence the success of *in ovo* probiotic strategies. Critical variables include strain selection, inoculum concentration, injection site accuracy, embryo viability, and compatibility with automated hatchery equipment [235]. Overdosing or inappropriate strain combinations may negatively affect hatchability or produce inconsistent immune responses. Therefore, precise standardization of dose and formulation is essential for safe large-scale implementation [273].

From a practical perspective, *in ovo* delivery offers the advantage of uniform administration at the hatchery level, potentially ensuring consistent early-life exposure across large flocks [234]. However, integration into commercial hatchery workflows requires validation of biosafety, equipment calibration, and cost–benefit feasibility. Moreover, the long-term persistence of immunomodulatory effects compared with conventional post-hatch oral supplementation remains an area requiring further longitudinal investigation [274].

## CHALLENGES AND LIMITATIONS OF PROBIOTIC USE

Probiotics can improve digestive health, modulate immunity, and enhance pathogen resistance in poultry, but their application faces multiple challenges [275]. Efficacy often varies depending on the strain, dosage, administration form, bird age, and breed, making standardized recommendations difficult [38]. Reproducibility across studies is limited by inconsistent experimental designs, endpoints, and reporting standards, highlighting the need for precision-based strategies tailored to flock genetics, baseline microbiota, and farm conditions [276].

Field applications introduce further variability. While controlled trials often show clear benefits, commercial farm outcomes are influenced by management practices, biosecurity, feed quality, environmental stress, and pathogen load [277]. This heterogeneity complicates generalization of results, emphasizing the importance of multi-site trials under standardized protocols [278].

Safety and regulatory considerations are critical. Certain probiotic strains may harbor transferable ARGs, requiring genomic screening to prevent potential horizontal gene transfer and AMR dissemination [279]. Strain authentication, virulence exclusion, and compliance with GRAS/QPS frameworks are necessary, while regulatory requirements vary across jurisdictions, affecting registration, labeling, and guidance for farmers [280]. Rare opportunistic infections have been reported in specific *Enterococcus* strains, underscoring the need for continuous post-market surveillance [281].

Stability and quality control are additional limitations. Non-spore-forming strains are sensitive to heat, oxygen, and acidic conditions, reducing viable cell counts and efficacy [282]. Batch-to-batch consistency, accurate CFU labeling, and shelf life management are essential, particularly under intensive production systems and variable storage conditions [283]. Delivery methods, including in-feed, in-water, and early-life *in ovo* administration, also face technical and operational challenges that require robust field validation [284].

Economic and practical constraints influence adoption. Cost-effectiveness depends on product price, inclusion rate, baseline farm performance, and disease pressure, while scalability, feed processing, and infrastructure limitations may hinder implementation. Farmer education and clear communication of scientific evidence are crucial for adoption and appropriate use [285].

Finally, future research should focus on standardized immune biomarkers, precision dosing, longitudinal ecological impacts on gut microbiota and AMR, and emerging delivery strategies. Integrating early-life interventions with microbiome-informed flock management may improve consistency and support the sustainable use of probiotics in post-AGP poultry production systems.

## IMPLICATIONS FOR THE POULTRY INDUSTRY

The use of probiotics in poultry plays a strategic role in the modern livestock industry, particularly in efforts to reduce or replace AGP use [37]. Probiotics can improve digestive health, modulate the immune system, and suppress pathogen growth, thus providing a safe, natural alternative to AGPs [286]. By implementing probiotics, farmers can maintain or even improve poultry production performance, including body weight, feed conversion efficiency, and product quality, without the risk of antibiotic residues in meat or eggs [21]. In several structured AGP replacement programs, probiotics have been incorporated as part of integrated health management systems combining improved biosecurity, optimized nutrition, and vaccination, enabling gradual reduction of in-feed antibiotics while maintaining performance and flock stability.

## MARKET TRENDS AND REGIONAL ADOPTION PATTERNS

Globally, the use of probiotics in poultry feed has increased substantially over the past decade, largely driven by regulatory restrictions on AGPs and growing consumer demand for antibiotic-free products [80]. Adoption rates are particularly high in regions where AGPs have been banned or heavily restricted, such as parts of Europe, while rapid market growth is also observed in Asia and Latin America due to expanding poultry production and export-oriented standards [287]. In contrast, adoption in some developing regions remains variable, influenced by product cost, regulatory frameworks, and access to technical support. These regional differences highlight the importance of economic and policy context in shaping probiotic implementation strategies [288].

## SHORT-TERM APPLICABLE STRATEGIES FOR INDUSTRY IMPLEMENTATION

In practical application, the success of probiotic use depends heavily on selecting the right strain, determining the optimal dosage, and using a stable dosage form [289]. Probiotics can be administered continuously through feed or drinking water, or combined with prebiotics to form synbiotics to achieve synergistic effects [290]. Administration from the early stages of poultry life can support lymphoid organ development, strengthen beneficial microbiota colonization, and accelerate the immune response to vaccines and pathogen exposure [291]. Importantly, integration with vaccination schedules represents a practical strategy, where probiotic supplementation is timed before and after live or inactivated vaccination to enhance antibody titers, mucosal IgA responses, and overall vaccine responsiveness while minimizing post-vaccination stress [292].

In the short-term, poultry producers can adopt evidence-based strategies such as early-life supplementation (particularly during the first week post-hatch), targeted administration during stress periods (e.g., vaccination, feed transition, environmental stress), and integration with biosecurity and nutritional management programs [208]. Routine monitoring of performance indicators (FCR, weight gain, mortality) can be used to evaluate practical effectiveness under farm conditions. These approaches are immediately applicable within existing production systems [293].

## ILLUSTRATIVE COMMERCIAL CASE EXAMPLES

Several commercial operations have reported measurable improvements following structured probiotic implementation programs. For example, broiler farms transitioning to antibiotic-free production systems have documented improvements in FCR and reduced enteric disease incidence after integrating multi-strain *Bacillus*- or *Lactobacillus*-based probiotics into feed throughout the production cycle [15]. Some AGP withdrawal programs have also reported reduced therapeutic antibiotic interventions and lower cumulative mortality rates after probiotic inclusion, particularly under moderate pathogen pressure [294]. In layer operations, continuous probiotic supplementation has been associated with improved egg production stability and shell quality, particularly under heat stress conditions [185]. While outcomes vary depending on management and environmental factors, these real-world experiences demonstrate that probiotic strategies can deliver tangible production and health benefits when properly implemented and monitored.

In addition to providing short-term benefits, probiotic administration also has long-term positive impacts on poultry health and productivity [193]. Continuous supplementation can increase resistance to infection, reduce the incidence of gastrointestinal diseases, and reduce oxidative stress [295]. These impacts are reflected in an extended productive lifespan, reduced mortality rates, and increased farm economic efficiency [169]. From a sustainability perspective, probiotic-based AGP replacement strategies contribute to reduced antimicrobial usage, mitigation of AMR selection pressure, lower flock mortality, and improved overall production efficiency, aligning poultry systems with One Health and responsible antibiotic stewardship principles [296]. Implementing a probiotic-based nutritional strategy supports more sustainable poultry production, is safe for consumers, and complies with international regulations restricting the use of antibiotics as growth promoters [297].

## LONG-TERM RESEARCH AND DEVELOPMENT GOALS

From a long-term perspective, further research is required to optimize strain selection through genomic and functional characterization, clarify strain-specific mechanisms of immune modulation, and develop precision dosing strategies based on host genetics and microbiota profiling [298]. Future studies should also focus on large-scale field validation trials, cost–benefit modeling under diverse production systems, and the development of more stable formulations with extended shelf life. Additionally, harmonization of regulatory frameworks and standardization of quality control protocols represent important long-term objectives to ensure global consistency in probiotic application [15]. These research directions will strengthen scientific understanding and enhance sustainable integration of probiotics into modern poultry production systems.

## EMERGING ANALYTICAL APPROACHES

Recent advances in multi-omics and computational biology provide new opportunities to move beyond descriptive mechanisms of probiotic action toward predictive and quantitative understanding. Metagenomics can profile gut microbiota composition and diversity, metabolomics can quantify bioactive metabolites like SCFAs, and transcriptomics enables monitoring of immune gene expression in response to probiotics [299]. By integrating these datasets, researchers can identify strain-specific effects, host–microbe interactions, and mechanistic pathways underlying immune modulation and pathogen resistance [300]. Table 5 provides a concise overview of major multi-omics tools used in poultry probiotic research, detailing their specific applications and illustrating how machine learning can leverage these datasets to generate predictive insights, such as forecasting immune enhancement or strain-specific colonization patterns [299–302].

Machine learning and artificial intelligence approaches have emerged as powerful tools for analyzing complex multi-omics datasets and predicting probiotic responses. For example, ML algorithms trained on 2025 poultry bioinformatics datasets can forecast optimal probiotic strain combinations to reduce Salmonella or enhance IgA/IgY responses [301]. Such models allow strain optimization under specific environmental or stress conditions, potentially improving feed efficiency and disease resilience. A conceptual example includes ML models predicting a 15% improvement in immunity through synergistic multi-strain formulations based on microbiota-metabolite associations [301].

**Table 5:** Summary of key multi-omics and computational tools used in poultry probiotic research, their applications, and predictive insights.

**Analytical tool**	**Main application in probiotic research**	**Example insight/predictive output**	**Relevance to precision poultry nutrition**	**References**
**Metagenomics**	Characterization of gut microbiota composition, taxonomic diversity, microbial community structure, and strain-specific colonization	Identify probiotic colonization dynamics, microbial community shifts, and pathogen suppression signatures (e.g., Salmonella exclusion)	Supports selection of probiotic strains based on microbiota compatibility, colonization potential, and microbial ecosystem stability	[299, 300]
**Metabolomics**	Quantification of short-chain fatty acids and other bioactive microbial metabolites, including acetate, propionate, butyrate, and antimicrobial peptides	Associate microbial metabolites with epithelial integrity, regulatory T cell differentiation, and anti-inflammatory immune responses	Facilitates metabolite-guided optimization of immune resilience, gut health, and feed efficiency	[299, 300]
**Transcriptomics**	Analysis of host immune gene expression, epithelial barrier function, and stress-responsive signaling pathways	Predict cytokine expression profiles (e.g., IL-10, IFN-γ, and IL-1β), tight junction gene regulation, and activation of immune pathways following probiotic supplementation	Enables host response-guided selection and optimization of probiotic formulations	[299, 300]
**Machine learning/artificial intelligence**	Integration of multi-omics datasets with phenotypic, immunological, microbiological, and production data	Predict immune responses, identify optimal probiotic combinations, model pathogen reduction, and forecast production performance	Supports precision probiotic design, individualized nutritional strategies, and environment-specific decision-making for poultry production	[301, 302]

By combining multi-omics profiling with AI/ML-driven prediction, this section highlights a novel, data-driven framework for precision probiotic development in poultry. This integrative approach advances the field beyond descriptive summaries and aligns with emerging 2025–2026 trends in computational biology, feed optimization, and pathogen-targeted probiotic design [302].

## FURTHER RESEARCH DIRECTIONS

Research on probiotics in poultry continues to offer substantial opportunities, particularly in strain-specific immunomodulation [210]. Future studies should focus on defining immune signatures, cytokine patterns, mucosal IgA, T cell polarization, and vaccine-associated antibody kinetics, to move beyond generalized efficacy claims. Multi-omics approaches (metagenomics, transcriptomics, metabolomics) can clarify interactions between probiotics, gut microbiota, and the avian immune system, revealing molecular pathways that influence gut health [303].

Beyond classical immune endpoints, probiotics may also modulate stress and behavior via the gut–brain axis. Evidence shows reductions in H/L ratios and corticosterone levels under heat or management stress, suggesting benefits for welfare, resilience, and performance. Integrating behavioral assays, stress biomarkers, and microbiome profiling can elucidate neuroimmune mechanisms in poultry [135].

Innovative strategies include recombinant probiotics and early-life interventions. Engineered strains can produce targeted bioactive molecules to enhance immunity and vaccination responses [304]. Early post-hatch supplementation may shape long-term gut–immune development and disease resilience. Complementary approaches like postbiotics and paraprobiotics, non-viable microbial components or metabolites, offer advantages in stability, safety, and precise dosing [305]. Comparative field studies are needed to evaluate efficacy, stability, cost-effectiveness, and regulatory feasibility of live probiotics, synbiotics, and non-viable alternatives [306].

Sustainability and One Health outcomes warrant attention. Probiotics may improve nutrient utilization, reduce ammonia emissions, and lower pathogen loads in litter, potentially mitigating environmental contamination and AMR. Life-cycle assessments and longitudinal farm-scale evaluations are needed to quantify these benefits [307].

Breed-specific and local strains represent another priority. Tailoring probiotics to genetic and physiological differences may optimize immune responses and microbiota colonization, while locally adapted strains can improve viability, safety, and cost-effectiveness [308].

**Figure 4:**
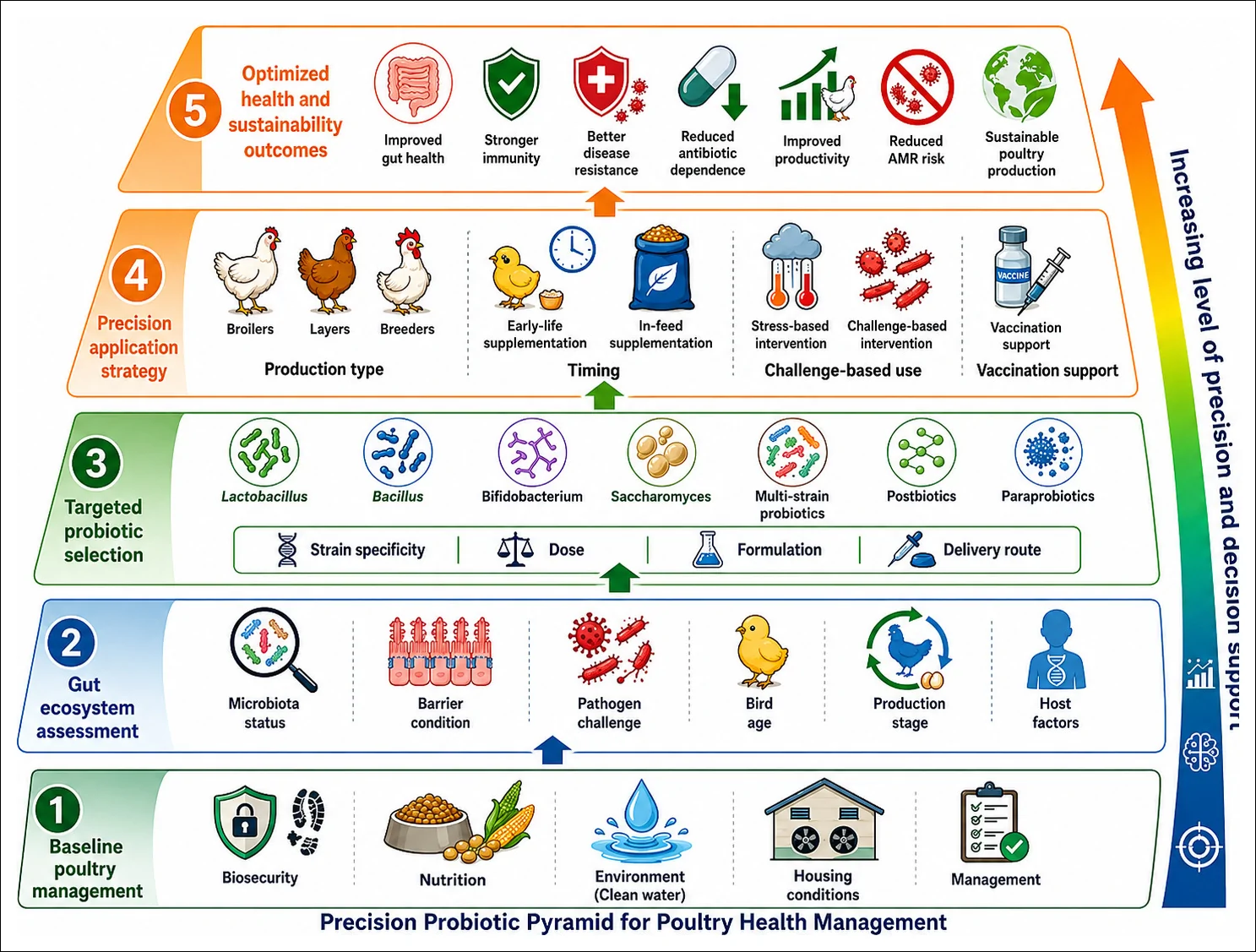
Precision Probiotic Pyramid for poultry health management. This schematic illustration was conceptually developed based on published evidence [303–310] and generated using artificial intelligence tools (ChatGPT 5.2), then subsequently modified by the authors.

Figure 4[303–310] illustrates the “Precision Probiotic Pyramid” as a conceptual framework for poultry health management. The base of the pyramid represents conventional single-strain probiotics, the middle layer includes synbiotics, postbiotics, and multi-strain formulations, while the apex integrates precision-designed probiotics guided by artificial intelligence, multi-omics approaches, and breed-specific data to maximize efficacy under stress or disease challenge conditions. Future innovations may involve engineered probiotics, targeted metabolite delivery, and predictive AI modeling, validated through large-scale, multi-site field trials.

From an industry perspective, priorities include scalable production, feed stability, cost–benefit validation, and integration with vaccination and antimicrobial stewardship [309]. From a research perspective, focus areas include reproducible trials, standardized immune biomarkers, precision dosing, and mechanistic validation of strain-specific effects to strengthen translational reliability [310].

## CONCLUSION

This integrative review synthesizes current evidence on the mechanistic roles of probiotics in modulating the gut–immune axis and their potential as sustainable alternatives to AGPs in poultry production. Key findings demonstrate that selected probiotic strains, particularly *Lactobacillus* spp., *Bacillus* spp., and *Bifidobacterium* spp., enhance innate immunity through macrophage activation and cytokine regulation, strengthen adaptive responses via increased mucosal IgA and systemic IgY production, improve lymphoid organ development, and reduce pathogen colonization through competitive exclusion and antimicrobial metabolite production. These immunomodulatory effects are associated with improved intestinal barrier integrity, balanced inflammatory responses, enhanced vaccine responsiveness, and better performance metrics under both controlled and field conditions. Postbiotics and paraprobiotics further expand options by offering greater stability and safety profiles while retaining bioactive benefits.

Probiotics provide a viable, natural strategy for supporting poultry health and productivity in the post-AGP era. Strategic implementation, through early-life or continuous supplementation, synbiotic combinations, and integration with vaccination programs, can reduce reliance on therapeutic antibiotics, lower pathogen loads (*Salmonella*, *Campylobacter*), improve feed efficiency, and enhance flock resilience to stress and disease. Commercial adoption should prioritize strain-specific selection, optimal dosing, stable delivery forms, and on-farm monitoring to maximize economic returns and ensure consistent outcomes across production systems.

This review offers a comprehensive, mechanism-driven synthesis that positions immune modulation as the central framework rather than a secondary outcome. It integrates molecular, cellular, organ-level, and production data, clearly distinguishes strain-specific effects, and bridges controlled research with practical industry applications, providing actionable guidance for veterinarians, nutritionists, and producers.

Considerable heterogeneity exists across studies due to differences in strains, dosages, experimental designs, bird types, and environmental conditions. Many mechanistic insights rely on *in vitro* or mammalian-derived data, with fewer direct avian molecular validations. Field reproducibility can be lower than in controlled trials, and long-term ecological impacts, cost–benefit analyses, and regulatory harmonization require further clarification.

Priority research directions include large-scale, multi-site field trials with standardized immune biomarkers, precision dosing guided by multi-omics and AI/ML approaches, development of recombinant and heat-stable formulations, and longitudinal evaluations of sustainability outcomes (AMR reduction, environmental impact). Breed-specific and locally adapted strains, early-life/*in ovo* interventions, and comparative studies of live probiotics versus postbiotics/paraprobiotics will further strengthen translational reliability and support climate-resilient poultry production.

In conclusion, probiotics represent a scientifically sound and practically viable tool for enhancing immune competence, disease resistance, and sustainable productivity in modern poultry systems. By shifting from generalized supplementation to precision, mechanism-based strategies, the industry can achieve meaningful reductions in antibiotic use while maintaining or improving flock health and performance. Continued research and evidence-based implementation will be essential to fully realize the potential of probiotics within a One Health framework for responsible and resilient poultry production.

## GENERATIVE ARTIFICIAL INTELLIGENCE DECLARATION

The authors used generative artificial intelligence tools to assist with language editing and preparation of selected sections of the manuscript. The generated content was critically reviewed, revised, and verified by the authors. All experimental design, data collection, analysis, interpretation, and conclusions were performed by the authors, who assume full responsibility for the content of the article. No artificial intelligence tool was credited as an author.

## AUTHORS’ CONTRIBUTIONS

ABY, WPL, BPP, and ARK: Drafted the manuscript. ML, ZNAR, KP, and MAA: Revised and edited the manuscript. EKS, MAF, and BA: Contributed to manuscript preparation and critically reviewed the manuscript. RZA, WW, and SR: Revised and edited the references. All authors have read and approved the final version of the manuscript.
